# Bio-Guided Fractionation Driven by In Vitro α-Amylase Inhibition Assays of Essential Oils Bearing Specialized Metabolites with Potential Hypoglycemic Activity

**DOI:** 10.3390/plants9091242

**Published:** 2020-09-21

**Authors:** Francesca Capetti, Cecilia Cagliero, Arianna Marengo, Carlo Bicchi, Patrizia Rubiolo, Barbara Sgorbini

**Affiliations:** Department of Drug Science and Technology, University of Turin, Via Pietro Giuria 9, 10125 Turin, Italy; francesca.capetti@unito.it (F.C.); cecilia.cagliero@unito.it (C.C.); arianna.marengo@unito.it (A.M.); carlo.bicchi@unito.it (C.B.); patrizia.rubiolo@unito.it (P.R.)

**Keywords:** essential oil, α-amylase inhibition, bio-guided fractionation, *Myristica fragrans*, *Eucalyptus radiata*

## Abstract

Type 2 diabetes mellitus (T2DM) is a metabolic disorder characterized by unpaired blood glycaemia maintenance. T2DM can be treated by inhibiting carbohydrate hydrolyzing enzymes (α-amylases and α-glucosidases) to decrease postprandial hyperglycemia. Acarbose and voglibose are inhibitors used in clinical practice. However, these drugs are associated with unpleasant gastrointestinal side effects. This study explores new α-amylase inhibitors deriving from plant volatile specialized metabolites. Sixty-two essential oils (EOs) from different plant species and botanical families were subjected to α-amylase in vitro enzymatic assay and chemically characterized using gas chromatography coupled to mass spectrometry. Several EOs were found to be potential α-amylase inhibitors, and *Eucalyptus radiata*, *Laurus nobilis*, and *Myristica* *fragrans* EOs displayed inhibitory capacities comparable to that of the positive control (i.e., acarbose). A bio-guided fractionation approach was adopted to isolate and identify the active fractions/compounds of *Eucalyptus radiata* and *Myristica fragrans* EOs. The bio-guided fractionation revealed that EOs α-amylase inhibitory activity is often the result of antagonist, additive, or synergistic interactions among their bioactive constituents and led to the identification of 1,8-cineole, 4-terpineol, α-terpineol, α-pinene, and β-pinene as bioactive compounds, also confirmed when they were tested singularly. These results demonstrate that EO oils are a promising source of potential α-amylase inhibitors.

## 1. Introduction

The term "diabetes mellitus" groups together a series of metabolic disorders characterized by hyperglycemia that is caused by the impairment of insulin secretion, action, or both. If not treated, chronic hyperglycemia, in association with diabetes, leads to long-term damage, dysfunction and organ failure especially in the eyes, kidneys, heart, nerves, and blood vessels [1]. Diabetes is classified into two broad etiopathogenetic categories: type 1 diabetes mellitus (T1DM) and type 2 diabetes mellitus (T2DM). T1DM is related to hyperglycemia caused by an absolute deficiency of insulin secretion and requires exogenous insulin administration if a patient is to survive. T2DM affects 90 to 95% of diabetic patients with impaired glucose tolerance that is related to diminished tissue response to insulin and is often associated with relative insulin deficiency. A common therapeutic approach for TD2M, especially for patients with unpaired post-prandial blood glucose excursion, is to inhibit the carbohydrate digestive enzymes (α-amylases and α-glucosidases) in order to delay glucose absorption and mitigate the post prandial increase in blood glucose [1]. α-Amylases (salivary and pancreatic α-amylases) are involved in the early stage of complex carbohydrate digestion, as they catalyze the hydrolysis of 1,4-α-glucosidic bonds in glucose units of long chain carbohydrates (i.e., amylose and amylopectin) forming maltose, maltotriose, and α-limit dextrin residues, which are then hydrolyzed into D-glucose monomers by α-glucosidases. Commonly prescribed α-glucosidase and α-amylase inhibitors (e.g., acarbose) are only partially effective at decreasing hyperglycemia [2], and present unpleasant gastrointestinal sides effects (i.e., meteorism, flatulence, and diarrhea) [3,4].

Medicinal plants have proven to be potentially promising in the treatment of T2DM. Hydroalcoholic and aqueous extracts that are rich in phenolic compounds, such as anthocyanins from *Ipomoea batatas* (L.) Poir. (Convolvulaceae) and *Pharbitis nil* (L.) Choisy (Convolvulaceae), and polyphenols from *Pinus pinaster* Aiton (Pinaceae), have effective inhibitory activity against intestinal α-glucosidase and α-amylases from human salivary glands [5,6,7]. 

Relatively less attention has been paid to volatile compounds (e.g., terpenoids) and to essential oils (EOs). They are complex mixtures of volatiles that are either obtained by steam distillation, dry distillation, or by mechanical means from a single plant species. Terpenoids are the most important group of specialized metabolites in EOs, and include, in particular, hemiterpenoids (C5), monoterpenoids (C10), and sesquiterpenoids (C15), whose volatility is compatible with the steam distillation process [8]. In addition to their use in the flavor and fragrance fields, EOs and their components are known for their wide range of biological activities. Specific EOs/EO components have proven to be effective for use as adjuvants in the treatment of a broad range of pathological conditions, including functional dyspnea (*Mentha* x *piperita* and *Carum carvi* EOs) [9], irritable bowel syndrome (*Mentha* x *piperita* EO) [10], respiratory disorders (i.e., menthol and 1,8-cineole) [10,11,12], gastroesophageal reflux (i.e., limonene) [13], and the dissolution of hepatic and renal stones (i.e., Rowatinex®, a commercial mixture of α-pinene, β-pinene, camphene, borneol, anethol, fenchone, and 1,8-cineole) [14,15]. To the best of the authors knowledge, very few studies have been performed on the effect of EOs on T2DM, and those reported in the literature mainly focus on the inhibitory activity of individual EO components rather than on an EO as a whole [16,17,18]. 

This study deals with the preliminary screening of 62 hydrodistilled EOs from different botanical families and species, in order to evaluate their potential hypoglycemic activity (i.e., α-amylase inhibition activity). Bio-guided fractionation, based on an in vitro α-amylase inhibition assay, was also performed to isolate the bioactive fractions and/or to identify the EO components with the most significant activity. Gas chromatography coupled to mass spectrometry (GC-MS) was used to identify the compounds that are characteristic of both the EOs and the isolated fractions, and that are responsible for their α-amylase inhibition activity.

## 2. Results and Discussion

**Figure 1:**
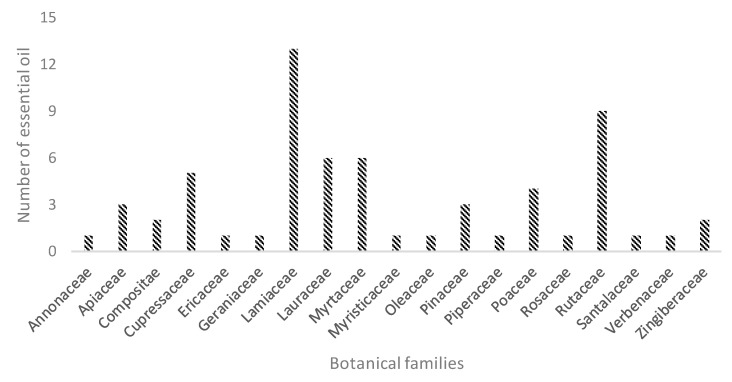
Distribution of the investigated EOs across different botanical families.

Table 1 reports a list of the 62 EOs screened in this study. They belong to different botanical families (i.e., Annonaceae, Apiaceae, Compositae, Cupressaceae, Ericaceae, Geraniaceae, Lamiaceae, Lauraceae, Myristicaceae, Myrtaceae, Oleaceae, Pinaceae, Piperaceae, Poaceae, Rosaceae, Rutaceae, Santalaceae, Verbenaceae, Zingiberaceae), as described in Figure 1.

### 2.1. Optimization of the In Vitro Enzymatic Test

EOs are poorly soluble in water, and, as in vitro enzymatic tests are carried out in an aqueous buffer solution, the choice of the solvent to be used as a “bridge” to solubilize the EO in the mixture test is the first aspect to be optimized, while avoiding interference with 1) enzymatic activity, 2) UV absorption, and 3) pH variation. Three solvents were tested as solubilizing solvents: ethyl acetate was the only one giving a clear solution when adding 50 μL of the EO solution, while methanol, and ethanol gave opalescent solutions. Absorbance at 540 nm and the pH variation of a 1 mL of buffer solution containing 50 μL of ethyl acetate were measured to evaluate any possible interference. Absorbance was found to be very low (0.080 ± 0.0003, *n* = 5) and pH variation negligible (from 7.00 to 7.13) and ethyl acetate was therefore selected as the “bridge” solvent. The required EO final concentration in the reaction mixture was set at 0.670 mgmL^−1^ to match the half maximal inhibitory concentration (IC_50_) of acarbose (0.601 ± 0.0673 mg mL^−1^). To minimize the amount of ethyl acetate in the reaction mixture, thus avoiding partial interference with the enzymatic activity, 10 μL of a 200 mg mL^−1^ solutions in ethyl acetate of each investigated EO were added to the enzymatic assay reaction mixture.

The high volatility of EO components is the second aspect to be considered. The parameters to be defined in this respect are the headspace volume of the vial containing the reaction mixture, and whether the biological test must be run in sealed vials. The experiments demonstrated that the reaction must be carried out in closed vials to minimize the headspace and limit the loss of bioactive volatile compounds in the headspace/environment (data not shown). A 4.0 mL vial was used for all of the in vitro inhibition experiments as the total volume of the reaction mixture was 3.0 mL.

### 2.2. In Vitro α-Amylase Inhibition Test

The inhibitory activity against α-amylase of the EOs listed in Table 1 was tested in vitro using a spectrophotometric assay (carried out in triplicate for each EOs) to evaluate their antidiabetic/hypoglycemic properties. Acarbose was chosen as the α-amylase inhibitor positive reference. The percentage α-amylase inhibition data are reported in Table 1. They were determined by measuring the difference in absorbance as reported in the Experimental Section (see par. Section 3.2). The most active EOs were *Eucalyptus radiata*, *Myristica fragrans,* and *Laurus nobilis*, which are characterized by inhibition activities higher than, or very similar to, that of acarbose, 65%, 59%, and 51%, respectively. Within the Lauraceae family, in addition to laurel EO, *Cinnamomum camphora* (20%) showed interesting activity, although it was lower than that of acarbose. Similar considerations can be made for other botanical families, with several species presenting interesting inhibition activity, i.e., Myrtaceae (*Corymbia citriodora* 44%, *Eucalyptus globulus* 34%, *Melaleuca viridiflora* 28%, *Myrtus communis* 20%), Lamiaceae (*Mentha arvensis* 39%, *Mentha* x *piperita* 33%, and 40% for leaf and leaf-twig EOs, respectively), and Compositae (*Artemisia vulgaris* 48% and *Matricaria chamomilla* 32%).

The percentage of inhibition of the most active EOs was confirmed by comparing the amounts of maltose produced during the enzymatic reaction in absence and in presence of the possible inhibitor. A calibration curve was built by plotting the optical density at 540 nm of solutions of known concentration of maltose previously reacted with 3,5-dinitrosalicylic acid (DNSA) in function of maltose concentration. Knowing the final absorbance at 540 nm, the effective amount of maltose liberated during the enzymatic reaction was determined by interpolation from the regression line of A 540nm on maltose concentration (y = −0.048 + 1.4x, r^2^ = 0.9985, 95% confidence interval for the slope 1.3 < β < 1.5; SEb = 0.10, t0.005(2), 3 = 3.18). The results obtained using the two approaches were compared, in particular for the three most active EOs, adopted as reference, i.e., laurel, nutmeg, and eucalyptus (Table 2). Percentage inhibition values were found to match perfectly, as the variation coefficient (% CV) was always below 0.8%. 

Twenty-three EOs belonging to different botanical families were found to be inactive in inhibiting α-amylase at the tested concentration under the applied experimental conditions: *Cinnamomum zeylanicum* and *Cinnamomum cassia*, several species belonging to Lamiaceae (i.e., lemon balm, clary sage, thyme, and patchouli), fennel and clove, among others. 

### 2.3. Essential Oil Composition

All EOs, both active and inactive, were analyzed using gas chromatography, with flame ionization (FID) and mass spectrometry (MS) detectors, to characterize their composition and evaluate the relative percentage abundance of hydrocarbons and oxygenated compounds for correlation with the α-amylase inhibition activity. Table 3 reports the percentage of hydrocarbons and oxygenated compounds for each active EO, together with the most abundant compounds that characterize their composition. 

The total composition of all investigated EOs is reported in the Supplementary Material (Tables S1–S6). Few EOs showed similar percentages for both hydrocarbon and oxygenated compounds: *Cinnamomum camphora*, *Citrus limon*, *Melaleuca alternifolia*, *Myrtus communis*, and *Origanum majorana*. Only eight EOs are characterized by high percentages (above 70%) of hydrocarbons: *Citrus limon*, *Citrus medica*, *Citrus nobilis*, *Citrus × aurantium*, *Cupressus sempervirens*, *Juniperus communis*, *Juniperus virginiana*, and *Myristica fragrans*. The others mostly consisted of oxygenated compounds (from 62.2% of *Carum carvi* to 99.1% of *Mentha* × *piperita*). 

### 2.4. Bio-Guided Assay Fractionation of Eucalyptus radiata and Myristica fragrans

*Eucalyptus radiata* and *Myristica fragrans* were selected from the most active EOs for further bio-guided assay fractionation; the first is representative of a class of EOs with a composition that is prevalently oxygenated monoterpenes, and the latter of a class of EOs that mainly consists of terpenic hydrocarbons. While inhibiting α-amylase to nearly the same extent, *Eucalyptus radiata* and *Myristica fragrans* EOs have significantly different chemical compositions, and their bioassay-oriented fractionation should thus provide good insight into the components that may be responsible for their biological activity.

The pure oxygenated and hydrocarbon fractions of the investigated EOs were isolated using automated flash chromatography and were individually subjected to α-amylase inhibitory assays. The amount of the EO (960 mg) to be fractionated was selected so that a suitable amount of each fraction could be recovered for the next biological testing. 

**Figure 2:**
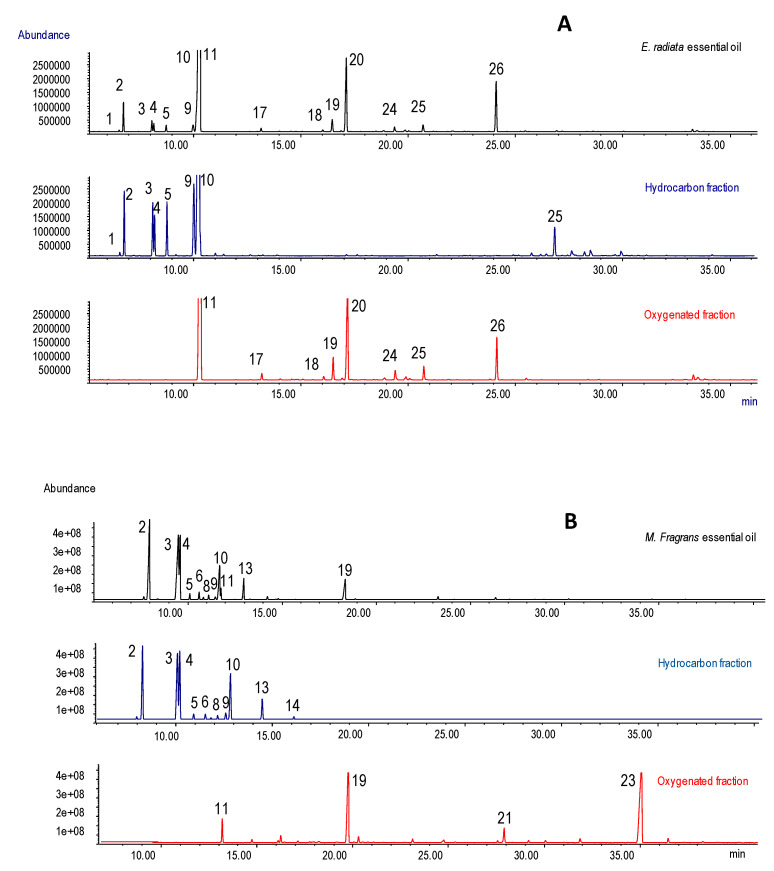
Gas chromatography coupled to mass spectrometry (GC-MS) profiles of the total EO together with those of the hydrocarbon and oxygenated fractions for *Eucalyptus radiata* (**A**) and *Myristica fragrans* (**B**) EOs. For identification see Table 4.

Flash chromatography separations were carried out on pre-packed 50 μm silica gel cartridges using a gradient of 0–20% ethyl-acetate in petroleum ether. All of the hydrocarbons eluted during the isocratic step of the gradient with 100% petroleum ether, and the complete fractionation of the pure material was completed within 20 minutes, regardless of the EO being treated. The yields of the oxygenated and hydrocarbon fractions were 123.6 mg and 718.1 mg, respectively, for the *Myristica fragrans* EO, and 744.7 mg and 82.4 mg, respectively, for *Eucalyptus radiata*. Each isolated fraction was analyzed using GC-MS and GC-FID to assess the chemical composition of the fraction and its chemical homogeneity. Figure 2 reports the GC-MS patterns of the total *Eucalyptus radiata* and *Myristica fragrans* EO together with those of the corresponding hydrocarbon and oxygenated fractions. Table 4 reports the composition of total *Eucalyptus radiata* and *Myristica fragrans* EOs, and of their hydrocarbon and oxygenated fractions, as obtained by flash chromatography.

The isolated oxygenated and hydrocarbon fractions for both EOs were tested separately for their α-amylase inhibitory activity at the same concentration as the original EO. Only the oxygenated fraction of *Eucalyptus radiata* EO inhibited α-amylase by 40%, while the hydrocarbon fraction was fully inactive. The oxygenated fraction of *Eucalyptus radiata* mainly consists of 1,8-cineole (68.9%) and α-terpineol (16.0%), which were found to be responsible for its α-amylase inhibitory activity. These compounds inhibited α-amylase by 31 ± 2 (IC_50_ 1.08 ± 0.0432 mg mL^−1^) and 33 ± 1% (IC_50_ 1.01 ± 0.0221 mg mL^−1^), respectively, when tested separately at a concentration of 0.670 mg mL^−1^. These values are quantitatively close to that of acarbose (IC_50_ 0.670 mg mL^−1^). Considering the relative percentage abundance of 1,8-cineole and α-terpineol in the oxygenated fraction of *Eucalyptus radiata* EO, and their respective inhibitory activities, the bioactivity of *Eucalyptus radiata* oxygenated fractions seems to be the result of synergic interactions between the two identified bioactive compounds. The observed bioactivity of 1,8-cineole agrees with the data available in the scientific literature [18,19]. However, a proper comparison was not possible due to the absence of the positive control (i.e., acarbose) in the referenced publications. The bioactivity of other tested EOs, namely *Cinnamomum camphora*, *Eucalyptus globulus*, *Hyssopus officinalis*, *Laurus nobilis,* and *Melaleuca viridiflora*, can thus partially be ascribed to the presence of 1,8-cineole, which is the major compound in all of the above mentioned EOs. However, linear regression analyses (data not shown) did not reveal a significant positive linear relationship between the amount of 1,8-cineole in an EO and the observed percentage of α-amylase inhibition. This is probably because of combination effects with other EO components having an influence on 1,8-cineole overall activity. 

The hydrocarbon and oxygenated fractions of *Myristica fragrans* EO inhibited α-amylase by 11.1% and 15.0%, respectively. Unlike *Eucalyptus radiata* EO, the chemical composition of this EO mainly consists of hydrocarbons, namely sabinene, α-pinene, β-pinene, and limonene, whose relative percentage areas are 27.0%, 23.0%, 13.0%, and 10.0%, respectively, and that are present in the corresponding isolated fraction in similar percentages (i.e., 26.3%, 21.8%, 20.4%, and 15.3%, respectively). When tested as pure standards, at a concentration of 0.670 mg mL^−1^, sabinene and limonene were inactive, while α- and β-pinene inhibited enzymatic activity by 32 ± 1% (IC_50_ 1.05 ± 0.0252 mg mL^−1^) and 29 ± 1% (IC_50_ 1.17 ± 0.0233 mg mL^−1^), respectively. This result suggests that they are responsible for the observed bioactivity of the hydrocarbon fraction, but that their contribution is only partial compared to that of the whole EO. The oxygenated fraction mainly contains 4-terpineol which, similarly to its structural isomer α-terpineol, inhibits α-amylase by 40 ± 2% (IC_50_ 0.838 ± 0.0335 mg mL^−1^) when tested individually at a concentration of 0.670 mg mL^−1^. Despite the evidence that has emerged from these experiments, further studies into the mechanism of enzymatic inhibition of both *Myristica fragrans* EO and its respective bioactive components are required to understand how they influence the overall EO activity.

The results obtained from the bioassay-oriented fractionation of *Myristica fragrans* EO have provided indications that allow us to, at least partially, understand the inhibition percentage results of other screened EOs. In addition to *Myristica fragrans*, the investigated EOs that contain high amounts of α and β-pinene are *Cupressus sempervirens*, *Juniperus communis*, *Pinus mugo,* and *Pinus sylvestris* EOs. However, unexpectedly, only the *Myristica fragrans* and *Cupressus sempervirens* EOs significantly inhibited α-amylase, while the others were inactive. In terms of chemical composition, *Myristica fragrans* and *Cupressus sempervirens* EOs mainly differ from the others because they are both free from *trans*-β-caryophyllene. Pearson’s correlation coefficient was thus measured to define whether an association exists between the presence of *trans*-β-caryophyllene and biological activity. The results suggest that the variables are indeed negatively related (r = −0.4583), and the correlation was sufficiently high to warrant the rejection of the null hypothesis of zero correlation (t = −2.871, df = 31, *p*-value = 0.007).

### 2.5. Data Precision

The precision of the in vitro α-amylase inhibition test was evaluated in terms of repeatability (by repeating the enzymatic inhibition assay three times in the same day) and intermediate precision (by repeating the enzymatic inhibition assay three times every four weeks over a period of six months). Results were very satisfactory and never exceeded 8.1% for repeatability and 11.8% for intermediate precision. Table 5 reports the percentage relative standard deviation (RSD%) for inhibition tests carried out with acarbose (reference) and with laurel, eucalyptus, and nutmeg EOs. Similar precision values were obtained for all the tested EOs.

## 3. Materials and Methods 

### 3.1. Reagents

Sixty-one EOs from different botanically authenticated species were supplied by Witt Italia SpA. *Mentha x piperita* EO (leaves) was obtained from fresh leaves (provided by Dr. Franco Chialva, Chialvamenta), and submitted to steam distillation. Table 1 reports the list of the EOs investigated, including botanical and common name, botanical family, and the part of the plant from which the EO was obtained. At least three different batches were considered for each EO. Pure standard samples of 4-terpineol, α-terpineol, 1,8-cineole, α-pinene, β-pinene, sabinene and limonene (purity > 98%) were purchased from Merck. The solvents were all HPLC-grade and obtained from Carlo Erba. Phosphate saline buffer, α-amylase from Aspergillus oryzae, maltose, acarbose, potato starch, and 3,5-dinitrosalicilic acid were also obtained from Merck.

### 3.2. In Vitro α-Amylase Inhibition Test 

The in vitro inhibition test adopted in this study was modified from that of Sigma-Aldrich [20]. Three different solvents (i.e., methanol, ethanol, and ethyl acetate) were tested to select the best one to be used as “bridge” to solubilize the EOs into the test mixture. 15 mg mL^−1^ solutions of tangerine and chamomile EOs were prepared for each investigated solvent. For each solution, 50 μL were added to 1 mL of buffer solution and UV absorption at 540 nm and pH variation were assessed. The enzyme solution was prepared daily at a concentration of 33.3 μg/mL in a 0.02 M phosphate buffer (pH 6.9). The starch solution (1% w/v) was obtained by stirring 1 g of potato starch in the same buffer at 90 °C for 15 min. Then, 200 mL of the 3,5-dinitrosalicylic reagent was prepared as follows: first, 2.18 g of 3,5-dinitrosalicylic acid (DNSA) was dissolved in 40 mL NaOH 2 M and 100 mL of deionized water; 60 g of potassium citrate (Rochelle salt) was then added and the volume was made up to 200 mL with deionized water; the solution was finally heated to 40 °C to dissolve the potassium citrate. The reactant solution was stored in the dark and protected from CO_2_ [21]. A 200 mg mL^−1^ solution in ethyl acetate was prepared and stored at 4 °C for each investigated EO, each standard sample, each isolated fraction, and for acarbose. 

Sample mixtures were prepared as follows: 10 μL of each EO solution (corresponding to an absolute amount of 2 mg) was added to 1 mL of enzyme solution (corresponding to 1 unit of α-amylase) and pre-incubated for 5 min at 25 °C under constant stirring. Then, 1 mL of the starch solution was added and incubated for 3 min at 25 °C. Finally, 1 mL of the DNSA solution was added and left to react for 15 min at 90 °C. The mixture was then cooled and diluted with 9 mL of deionized water and the absorbance was measured at 540 nm. The standard test containing the non-inhibited enzyme was prepared as described above, with 10 µL of pure ethyl acetate instead of the EO solution. The final concentration of EOs and acarbose in the reaction mixture was 0.67 mg mL^−1^ (i.e., 2 mg in terms of absolute amount), which corresponds to IC_50_ for acarbose. 

The α-amylase inhibition activity of each investigated EO was measured using two different approaches. The first approach (Method A) relies on difference in absorbance and α-amylase inhibition activity is measured by applying the following Equation (1):

(1) $$\%\;Inhibition=\;\frac{\left(A_{Standard}-\;A_{Standard\;blank}\right)-\;\left(A_{Sample}-A_{Sample\;blank}\right)}{\left(A_{Standard}-\;A_{Standard\;blank}\right)}\;\times 100$$

where *A_Standard_* is the absorbance at 540 nm when the enzyme was not inhibited, *A_Standard blank_* is the absorbance of the standard blank solution containing the same reagents as the standard mixture, but with 1 mL of buffer instead of the enzyme, *A_Sample_* is the absorbance at 540 nm in the presence of the investigated inhibitor, and *A_Sample blank_* is the absorbance of the sample blank solution prepared with the same reagents as the sample mixture, including the 10 uL of the EO solution, but with 1 mL of buffer instead of the enzyme. Each blank underwent the same steps as the corresponding sample. A blank of each EO was performed to account for possible variations in absorbance due to differences in EO chemical composition. Acarbose was used as the positive reference; positive reference inhibition tests were carried out using 10 μL of acarbose solution at a concentration of 200 mgmL^−1^ in ethyl acetate (corresponding to an absolute amount of 2 mg).

The second approach (Method B) measures amylase inhibition activity considering the differences in the amount of maltose generated by α-amylase activity via the reduction of 3,5-dinitrosalicylic acid to 3-amino-5-nitrosalicylic acid, by applying the following Formula (2):

(2) $$\%\;Inhibition=\;100-\;\frac{C_{maltose\;sample}}{C_{maltose\;Standard}}\;\times \;100$$

where *C_maltose sample_* is the concentration of maltose produced by α-amylase in the presence of EO or acarbose, and *C_maltose Standard_* is the concentration of maltose produced by the non-inhibited α-amylase.

### 3.3. Maltose Calibration Curve

Maltose solutions were prepared in 0.02 M phosphate buffer (pH 6.9) at seven different concentrations: 1.0, 0.75, 0.50, 0.25, 0.20, 0.15, and 0.10 mg mL^−1^. Then, 1.0 mL of each solution was mixed with 1.0 mL of buffer solution and 1 mL of the DNSA solution and the mixture was left to react for 15 min at 90 °C. Once the reaction was finished, the mixture was cooled and diluted with 9 mL of deionized water and the absorbance was read at 540 nm. The maltose calibration curve was built by plotting the absorbance at 540 nm as a function of maltose concentration (mg mL^−1^).

### 3.4. Flash Column Chromatography

EO fractionation was carried out on a flash column chromatography system PuriFlash 450 by Sepachrom, equipped with both UV and Evaporative Light Scattering (ELSD) detectors. Stationary phase: Sepachrom silica Daily 50 μm; mobile phase: petrolether (A) and ethyl acetate (B), flow 25 mL min^−1^. Linear gradient elution was adopted from 100% of A to 80% of A and 20% of B in 20 min.

### 3.5. Analysis Conditions

GC-MS analyses were carried out using a Gerstel MPS-2 multipurpose sampler installed on a Shimadzu 2010 GC unit coupled to a Shimadzu QP2010 Mass spectrometer. GC conditions: injector temperature: 280 °C, injection mode: split, ratio: 1/20; carrier gas: helium, flow rate: 1 mL min^−1^; column: Mega SE 52 (95% polydimethylsiloxane, 5% phenyl) 25 m × 0.25 mm dc × 0.25 μm df, from MEGA. Temperature program: from 50 °C (1 min) to 250 °C (5 min) at 3 °C min^−1^. MSD conditions: MS operated in EI mode (70 eV), scan range: 35 to 350 amu; dwell time 40 ms, ion source temperature: 230 °C; quadrupole temperature: 150 °C; transfer line temperature: 280 °C. EO markers were identified by comparing both their linear retention indices (LRIs), calculated versus a C9-C25 hydrocarbon mixture, and their mass spectra against those of authentic samples, or from commercially available mass spectral libraries (Adams, 2007).

GC-FID analyses were carried out on the previously-described Shimadzu 2010 GC unit. GC conditions: injector temperature: 280 °C, injection mode: split, ratio: 1/20; carrier gas: hydrogen, flow rate: 1 mL min^−1^; column: Mega SE 52 (95% polydimethylsiloxane, 5% phenyl) 25 m × 0.25 mm dc × 0.25 μm df, from MEGA. Temperature program: from 50 °C (1 min) to 250 °C (5 min) at 3 °C min^−1^. Relative percentage composition of the analyzed EOs was determined using GC-FID peak areas with response factors [22,23].

## 4. Conclusions

This study explores the α-amylase inhibition ability of 62 EOs of different composition and belonging to different plant families. Under the applied experimental conditions, three EOs showed high inhibitory capacity (*Eucalyptus radiata*, *Laurus nobilis,* and *Myristica fragrans*) that was comparable or slightly higher than that of acarbose, which was chosen as the positive control. The results showed that the EO components seem to act synergistically, as the total EO percentage inhibition activity is higher than those of both the combined isolated fractions and pure compounds. Moreover, an interesting number of both hydrocarbon and oxygenated compounds. were characterized as good α-amylase inhibitors, with percentage inhibitions of about 30% (i.e., 1,8-cineole, 4-terpineol, α-terpineol α-pinene, and β-pinene). These results demonstrate that EO oils are a promising source of potential α-amylase inhibitors.

## Figures and Tables

**Table 1:** List of the investigated essential oils (EOs), the part of the plant used to obtain them, botanical and common names, and percentage of α-amylase inhibition activity measured by absorbance differences (Method A).

Species	Family	Common Name	Plant Part	% Inhibition Activity	Standard Deviation
*Artemisia vulgaris* L.	Compositae	Mugwort	Leaf/Flower	48	3
*Cananga odorata* (Lam.) Hook.f. and Thomson	Annonaceae	Ylang-ylang	Flower	no activity	-
*Carum carvi* L.	Apiaceae	Caraway	Seed	17	2
*Cedrus atlantica* (Endl.) Manetti ex Carrière	Pinaceae	Cedar wood	Wood	no activity	-
*Chrysopogon zizanioides* (L.) Roberty	Poaceae	Vetiver	Root	no activity	-
*Cinnamomum zeylanicum* Nees	Lauraceae	Cinnamon leaf	Leaf	no activity	-
*Cinnamomum zeylanicum* Nees	Lauraceae	Cinnamon bark	Bark	no activity	-
*Cinnamomum camphora* (L.) J.Presl	Lauraceae	Camphor	Wood	20	2
*Cinnamomum cassia* (L.) J.Presl	Lauraceae	Cinnamon bark	Bark	no activity	-
*Citrus × aurantium* L.	Rutaceae	Bitter orange	Fruit peel	23	5
*Citrus × aurantium* L.	Rutaceae	Neroli	Flower	20	1
*Citrus × aurantium* L.	Rutaceae	Petitgrain	Leaf	4	1
*Citrus bergamia* Risso et Poiteau	Rutaceae	Bergamot	Fruit peel	16	3
*Citrus limon* (L.) Osbeck	Rutaceae	Lemon	Fruit peel	15	2
*Citrus medica* L.	Rutaceae	Finger citron	Fruit peel	14	4
*Citrus nobilis* Lour.	Rutaceae	Mandarin	Fruit peel	no activity	-
*Citrus paradisi* Macfad.	Rutaceae	Grapefruit	Fruit peel	no activity	-
*Citrus sinensis (L.)* Osbeck	Rutaceae	Sweet orange	Fruit peel	14	3
*Corymbia citriodora* (Hook.) K.D.Hill and L.A.S.Johnson	Myrtaceae	Eucalyptus. lemon-scented	Leaf	44	5
*Cupressus sempervirens* L.	Cupressaceae	Cypress	Leaf/Twig	17	1
*Cymbopogon martini* (Roxb.) W.Watson.	Poaceae	Palmarosa	Leaf	no activity	-
*Cymbopogon nardus* (L.) Rendle	Poaceae	Citronella Ceylon	Leaf	22	2
*Cymbopogon schoenanthus* (L.) Spreng.	Poaceae	Lemongrass	Leaf	7	2
*Elettaria cardamomum* (L.) Maton	Zingiberaceae	Cardamom	Seed	10	3
*Eucalyptus globulus* Labill.	Myrtaceae	Eucalyptus	Leaf	34	3
*Eucalyptus radiata* A.Cunn. ex DC.	Myrtaceae		Leaf	65	4
*Foeniculum vulgare* Mill.	Apiaceae	Fennel	Fruit	no activity	-
*Gaultheria procumbens* L.	Ericaceae	Wintergreen	Leaf	no activity	-
*Hyssopus officinalis* L.	Lamiaceae	Hyssop	Leaf	18	1
*Jasminum officinale* L.	Oleaceae	Jasmine	Flowers	no activity	-
*Juniperus communis* L.	Cupressaceae	Juniper berry	Fruit	5	2
*Juniperus virginiana* L.	Cupressaceae	Cedarwood	Wood	15	2
*Laurus nobilis* L.	Lauraceae	Laurel	Leaf	50	4
*Lavandula angustifolia Mill. × L. latifolia Medik.*	Lamiaceae	Lavandin	Leaf	6	3
*Lavandula angustifolia* Mill.	Lamiaceae	Lavender	Leaf	5	2
*Matricaria chamomilla* L.	Compositae	Chamomile	Flowers	32	4
*Melaleuca alternifolia* (Maiden and Betche) Cheel	Myrtaceae	Tea tree	Leaf	14	2
*Melaleuca viridiflora* Sol. ex Gaertn.	Myrtaceae	Niaouli	Leaf	28	4
*Melissa officinalis* L.	Lamiaceae	Lemon balm	Leaf	no activity	-
*Mentha × piperita* L.	Lamiaceae	Peppermint	Leaf	33	3
*Mentha × piperita* L.	Lamiaceae	Peppermint	Leaf/twig	40	5
*Mentha arvensis* L.	Lamiaceae	Mint	Leaf	39	6
*Myristica fragrans Houtt.*	Myristicaceae	Nutmeg	Seed	59	4
*Myrtus communis* L.	Myrtaceae	Myrtle	Leaf	20	7
*Ocimum basilicum* L.	Lamiaceae	Basil	Leaf	14	5
*Origanum majorana* L.	Lamiaceae	Marjoram	Leaf	10	1
Origanum vulgare L.	Lamiaceae	Oregano	Leaf	9	4
*Pelargonium graveolens* L’Hér.	Geraniaceae	Geranium	Leaf	9	3
*Pimpinella anisum* L.	Apiaceae	Aniseed	Fruit	27	5
*Pinus mugo* Turra	Pinaceae	Pine needle	Leaf/Twig	no activity	-
*Pinus sylvestris* L.	Pinaceae	Pine sylvestris	Leaf/Twig	no activity	-
*Piper nigrum* L.	Piperaceae	Black pepper	Fruit	no activity	-
*Pogostemon cablin* (Blanco) Benth.	Lamiaceae	Patchouli	Leaf	no activity	-
*Rosa × damascena* Herrm.	Rosaceae	Rosa	Flower	no activity	-
*Salvia officinalis* L.	Lamiaceae	Sage. Dalmatian	Leaf	3	2
*Salvia sclarea* L.	Lamiaceae	Clary sage	Leaf/Flower	no activity	-
*Santalum album* L.	Santalaceae	Sandalwood	Wood	no activity	-
*Syzygium aromaticum* (L.) Merr. and L.M.Perry	Myrtaceae	Clove oil	Leaf/Buds	no activity	-
*Thuja occidentalis* L.	Cupressaceae	Cedar leaf	Leaf	7	5
*Thymus vulgaris* L.	Lamiaceae	Thyme	Leaf	no activity	-
*Verbena officinalis* L.	Verbenaceae	Vervain	Leaf	no activity	-
*Zingiber officinale* Roscoe	Zingiberaceae	Ginger	Rhizome	no activity	-

**Table 2:** Comparison between the percentage of inhibition activity determined by absorbance differences (Approach A) and that obtained by applying the maltose calibration curve (Approach B).

	% Inhibition Approach A	IC_50_^1^ (mg mL^−1^)	% Inhibition Approach B	IC_50_^1^ (mg mL^−1^)	% CV ^2^
*Eucalyptus radiata*	65 ± 3	1.53 ± 0.08	65 ± 4	1.54 ± 0.11	0.080
*Myristica fragrans*	59 ± 4	1.70 ± 0.13	58 ± 5	1.71 ± 0.17	0.780
*Laurus nobilis*	51 ± 8	1.98 ± 0.32	51 ± 5	1.98 ± 0.19	0.120
Acarbose	56 ± 6	1.80 ± 0.20	55 ± 6	1.80 ± 0.22	0.020

^1^ = Half-maximal inhibitory concentration, ^2^ = Coefficient of variation.

**Table 3:** List of the most abundant specialized metabolites and hydrocarbon and oxygenated compounds percentage composition of those EOs displaying α-amylase inhibitory activity. (The complete chemical composition of each investigated EO is reported in Table S1 of Supplementary Material). Bold highlighted the most abundant fraction.

Species	Hydrocarbon Compounds	Oxygenated Compounds	List of the Most Abundant Components
*Artemisia vulgaris* L	9.0	**91.0**	α-Thujone (47.4), Camphor (30.0), β-Thujone (7.80), Sabinene (3.90), Camphene (3.70)
*Carum carvi* L.	37.8	**62.2**	Carvone (59.6), Limonene (35.4), β-Myrcene (0.700), *cis*-Dihydroxy carvone (0.600), *trans*-Dihydroxy carvone (0.200)
*Cinnamomum camphora* (L.) J.Presl	**54.8**	**45.2**	1,8-Cineole (44.1), Limonene (17.4), *p*-Cymene (14.6), α-Terpinene (9.60), β-Pinene (7.60)
*Citrus × aurantium* L. (neroli)	**97.5**	2.5	Linalyl acetate (41.4), Linalool (28.5), Limonene (11.4), β-Pinene (7.60), *trans*-β-Ocimene (2.60)
*Citrus × aurantium* L.	23.9	**76.1**	Limonene (90.2), β -Myrcene (3.70), Linalyl acetate (1.60), α-Pinene (0.900), Sabinene (0.500)
*Citrus × aurantium* L. (Petit grain)	7.4	**92.6**	Linalyl acetate (56.8), Linalool (24.4), α-Terpineol (5.60), Geranyl acetate (3.40), Neryl acetate (1.80)
*Citrus bergamia* Risso et Poiteau	**52.0**	**48.0**	Linalyl acetate (34.1), Limonene (32.3), γ-Terpinene (7.80), β-Pinene (6.60), α-Pinene (1.00)
*Citrus limon* (L.) Osbeck	**97.2**	2.8	Limonene (71.9), β-Pinene (11.6), γ -Terpinene (7.90), α-Pinene (1.50), β-Myrcene (1.50)
*Citrus medica* L.	**72.5**	27.5	Limonene (54.9), Linalyl acetate (14.5), β-Pinene (9.10), Linalool (4.60), Geranial (4.60)
*Citrus nobilis* Lour.	**99.9**	0.1	Limonene (75.6), γ -Terpinene (14.5), α-Pinene (1.90), β-Pinene (1.10), β -Myrcene (1.00)
*Corymbia citriodora* (Hook.) K.D.Hill and L.A.S.Johnson	1.8	**98.2**	Citronellal (81.0), Neoisopulegol (7.10), Citronellol (6.00), Citronellyl acetate (1.50), 1,8-Cineole (0.800)
*Cupressus sempervirens* L.	**91.9**	8.1	α-Pinene (46.7), Δ-3-Carene (25.3), α-Terpinolene (4.20), Limonene (4.00), α-Terpinyl acetate (3.30)
*Cymbopogon nardus* (L.) Rendle	11.6	**88.4**	Citronellal (37.9), Geraniol (19.5), Citronellol (12.5), Limonene (7.20), Geranyl acetate (4.40)
*Cymbopogon schoenanthus* (L.) Spreng.	7.0	**93.0**	Geranial (38.2), Neral (32.5), Geraniol (7.30), Geranyl acetate (4.20), *trans*-β-Caryophyllene (2.90)
*Elettaria cardamomum* (L.) Maton	9.2	**90.8**	α-Terpinyl acetate (43.8), 1,8-Cineole (34.7), Linalyl acetate (6.00), Linalool (2.70), Limonene (2.20)
*Eucalyptus globulus* Labill.	16.9	**83.1**	1,8-Cineole (82.1), Limonene (6.80), γ-Terpinene (3.30), *p*-Cymene (3.10), α-Pinene (2.20)
*Eucalyptus radiata* A.Cunn. ex DC.	14.6	**84.9**	1,8-Cineole (75.1), α-Terpineol (7.6), Limonene (4.3) α-Terpinene (4.4) α-Pinene (2.7)
*Hyssopus officinalis* L.	34.8	**65.2**	1,8-Cineole (39.2), α-Pinene (7.10), Isopinocamphone (6.10), Sabinene (5.80), β-Pinene (5.60)
*Juniperus communis* L.	**95.4**	4.6	α-Pinene (35.9), β -Myrcene (14.2), Sabinene (8.4) Limonene (8.00), β-Pinene (5.40)
*Juniperus virginiana* L.	**99.7**	0.3	β -Himachalene (50.8), α -Himalachene (16.0), γ-Himalachene (10.0), δ-Cadinene (2.50), α -Chamigrene (2.00)
*Laurus nobilis* L.	20.5	**79.5**	1,8-Cineole (65.4), α-Terpinyl acetate (8.10), α-Pinene (6.4), Sabinene (5.10), β-Pinene (3.80)
*Lavandula angustifolia* Mill. *× L. latifolia* Medik.	8.9	**91.1**	Linalyl acetate (35.6), Linalool (27.1), Camphor (9.40), 1,8-Cineole (7.60), Borneol (3.40)
*Lavandula angustifolia* Mill.	13.8	**86.2**	Linalyl acetate (34.7), Linalool (27.9), *trans*-β-Caryophyllene (4.20), 4-Terpineol (3.70), Lavandulyl acetate (3.70)
*Litsea cubeba* (Lour.) Pers.	17.3	**82.7**	Geranial (42.4), Neral (34.6), Limonene (12.6), Sabinene (1.90), α-Pinene (1.20)
*Matricaria chamomilla* L.	36.0	**64.0**	α-Bisabolol oxide A (47.0), *trans*-β-Farnesene (24.0), α-Bisabolol oxide B (6.4), Chamazulene (2.60), Germacrene D (1.60)
*Melaleuca alternifolia* (Maiden and Betche) Cheel	**45.9**	**54.1**	4-Terpineol (44.1), γ-Terpinene (21.1), α-Terpinene (9.40), α-Terpinolene (3.20), 1,8-Cineole (3.20)
*Melaleuca viridiflora* Sol. ex Gaertn.	23.4	**76.6**	1,8-Cineole (64.9), Limonene (9.50), α-Pinene (6.20), α-Terpineol (4.20), Viridiflorol (2.40)
*Mentha × piperita* L. (leaf)	1.1	**98.9**	Menthol (48.2), Menthone (24.9), Isomenthone (13.3), Menthyl acetate (6.40), Neomenthol (2.00)
*Mentha × piperita* L. (Leaf/Twig)	0.9	**99.1**	Menthol (52.0), Menthone (23.0), Isomenthone (10.0), Menthyl acetate (4.60), Neomenthol (4.26)
*Mentha arvensis* L.	6.6	**93.4**	Menthol (40.2), Menthone (19.5), Isomenthone (8.00), Menthyl acetate (7.40), Neomenthol (5.20)
*Myristica fragrans* Houtt.	**85.2**	14.8	Sabinene (27.0), α-Pinene (23.0), β-Pinene (13.0), Limonene (10.0), 4-Terpineol (6.70)
*Myrtus communis* L.	**56.1**	43.9	Limonene (28.9), α-Pinene (15.1), Mirtenyl acetate (13.6), Linalool (13.50), Linalyl acetate (5.00)
*Ocimum basilicum* L	5.1	**94.9**	Estragole (88.4), 1,8-Cineole (3.40), α-*trans*-Bergamotene (2.30), trans-β-Ocimene (1.00), Linalool (0.600)
*Origanum majorana* L.	41.3	**58.7**	Linalool (34.0), 4-Terpineol (17.7), γ-Terpinene (10.8), α-Terpinene (7.00), Sabinene (5.80)
*Origanum vulgare L.*	28.8	**71.2**	Carvacrol (67.4), *p*-Cymene (12.2), γ-Terpinene (5.10), trans-β-Caryophyllene (4.70), Linalool (1.90)
*Pelargonium graveolens* L’Hér.	5.1	**94.9**	Citronellol (34.6), Geraniol (18.5), Citronellyl formate (9.50), Linalool (6.70), Isomenthone (5.10)
*Pimpinella anisum* L.	3.7	**96.3**	*trans*-Anethol (92.2), Limonene (2.1), Estragole (1.70), Foeniculin (.0700), Linalool (0.400)
*Rosmarinus officinalis* L.	35.1	**64.9**	1,8-Cineole (43.3), Camphor (18.1), α-Pinene (12.8), β-Pinene (9.50), *trans*-β-Caryophyllene (5.90)
*Salvia officinalis* L.	32.2	**67.8**	α-Thujone (22.5), Camphor (18.5), 1,8-Cineole (11.4), α-Humulene (7.20), β-Thujone (6.20),
*Syzygium aromaticum* (L.) Merr. and L.M.Perry	10.2	**89.8**	Eugenol (82.0), *trans*-β-Caryophyllene (9.10), Eugenyl acetate (7.10), α-Humulene (1.10), Caryophyllene oxide (0.300)

**Table 4:** Chemical composition of *Eucalyptus radiata* and *Myristica fragrans* EOs and that of their respective hydrocarbon and oxygenated fractions.

#	Compounds	*Eucalyptus radiata*			*Myristica fragrans*		
		Total EO	Hydrocarbon Fraction	Oxygenated Fraction	Total EO	Hydrocarbon Fraction	Oxygenated Fraction
1	α-Thujene	0.2	0.3	/	/	0.7	/
2	α-Pinene	2.7	6.2	/	23.0	21.8	/
3	Sabinene	1.0	5.7	/	27.0	26.3	/
4	β-Pinene	0.6	4.2	/	13.0	20.4	/
5	β-Mircene	0.4	5.8	/	1.1	1.7	/
6	α-Phellandrene	/	/	/	1.4	1.6	/
7	Δ-3-Carene	/	/	/	0,4	0.6	/
8	α-Terpinene	4.4	/	/	0.9	1.2	/
9	*p*-Cimene	0.6	11.3	/	0.7	2.1	/
10	Limonene	4.3	65.4	/	10.0	15.3	/
11	1,8-Cineole	75.1	/	69.8	1.8	/	5.0
12	*trans*-β-Ocimene	tr	0.2	/	0.05	0.1	/
13	γ-Terpinene	tr	0.1	/	4.9	6.6	/
14	α-Terpinolene	tr	0.1	/	0.6	1.1	/
15	*cis*-Sabinene Hydrate	/	/	/	/	/	0.7
16	*trans*-Sabinene Hydrate	/	/	/	/	/	0.5
17	Linalool	0.3	/	0.7	0,2	/	1.6
18	Linalyl propionate	0.2	/	0.4	/	/	/
19	4-Terpineol	1.0	/	1.7	6.7	/	45.4
20	α-Terpineol	7.6	/	16.0	0.2	/	1.7
21	Eugenol	/	/	/	0.5	/	4.5
22	Safrole	/	/	/	0.7	/	1.1
23	Myristicin	/	/	/	4.4	/	37.3
24	Neral	0.3	/	1.2	/	/	/
25	Geranial	0.4	/	1.6	/	/	/
26	α-Terpinyl acetate	0.3	/	5.0	/	/	1.8
27	*trans*-β-Caryophyllene	/	0.6	/	/	0.2	/

**Table 5:** Data precision expressed as percentage relative standard deviation (RSD%) for both repeatability (*n* = 3) and intermediate precision (*n* = 6). * Values represent the average of three assays.

	Repeatability (*n* = 3)			Intermediate Precision	
	% Inhibition	% RSD		% Inhibition *	% RSD
**Acarbose**	57	2	**Acarbose**	59	5
	55			57	
	56			53	
				57	
				55	
				56	
**Laurel**	54	8	**Laurel**	54	12
	51			53	
	46			52	
				56	
				44	
				42	
**Nutmeg**	56	4	**Nutmeg**	54	9
	61			63	
	60			60	
				50	
				65	
				60	
**Eucalyptus**	66	4	**Eucalyptus**	54	9
	68			70	
	62			60	
				62	
				67	
				69	

* Values represent the average of three assays.

## Supplementary Materials

The following are available online at https://www.mdpi.com/2223-7747/9/9/1242/s1. Tables S1–S6: total chemical composition (expressed as relative percentage area of each identified compound of the 62 investigated essential oils).

## References

[1] American Diabetes Association (2014). Diagnosis and Classification of Diabetes Mellitus. Diabetes Care.

[2] Bailey C.J., Pickup J., William G. (2003). New Approaches to the pharmacotherapy of diabetes. Textbook of Diabetes.

[3] Fujisawa T., Ikegami H., Inoue K., Kawabata Y., Ogihara T. (2005). Effect of two α-glucosidase inhibitors, voglibose and acarbose, on postprandial hyperglycemia correlates with subjective abdominal symptoms. Metabolism.

[4] Ríos J., Francini F., Schinella G. (2015). Natural Products for the Treatment of Type 2 Diabetes Mellitus. Planta Med..

[5] Bedekar A., Shah K., Koffas M. (2010). Natural products for type II diabetes treatment. Adv. Appl. Microbiol..

[6] Nelson-Dooley C., Della-Fera M., Hamrick M., Baile C. (2005). Novel Treatments for Obesity and Osteoporosis: Targeting Apoptotic Pathways in Adipocytes. Curr. Med. Chem..

[7] Matsui T., Ueda T., Oki T., Sugita K., Terahara N., Matsumoto K. (2001). α-glucosidase inhibitory action of natural acylated anthocyanins. 1. Survey of natural pigments with potent inhibitory activity. J. Agric. Food Chem..

[8] Can Başer K.H., Buchbauer G. (2015). Handbook of Essential Oils.

[9] Goerg K.J., Spilker T. (2003). Effect of peppermint oil and caraway oil on gastrointestinal motility in healthy volunteers: A pharmacodynamic study using simultaneous determination of gastric and gall-bladder emptying and orocaecal transit time. Aliment Pharmacol. Ther..

[10] Cappello G., Spezzaferro M., Grossi L., Manzoli L., Marzio L. (2007). Peppermint oil (Mintoil®) in the treatment of irritable bowel syndrome: A prospective double blind placebo-controlled randomized trial. Dig. Liver Dis..

[11] Kenia P., Houghton T., Beardsmore C. (2008). Does inhaling menthol affect nasal patency or cough?. Pediatr. Pulmonol..

[12] Kehrl W., Sonnemann U., Dethlefsen U. (2004). Therapy for Acute Nonpurulent Rhinosinusitis with Cineole: Results of a Double-Blind, Randomized, Placebo-Controlled Trial. Laryngoscope.

[13] Wilkins J.S. (2002). Method for Treating Gastrointestinal Disorders. US Patent.

[14] Kim D.H., Goh H.J., Lee H.W., Kim K.S., Kim Y.T., Moon H.S., Lee S.W., Park S.Y. (2014). The effect of terpene combination on ureter calculus expulsion after extracorporeal shock wave lithotripsy. Korean J. Urol..

[15] Romics I., Siller G., Kohnen R., Mavrogenis S., Varga J., Holman E. (2011). A Special Terpene Combination (Rowatinex®) Improves Stone Clearance after Extracorporeal Shockwave Lithotripsy in Urolithiasis Patients: Results of a Placebo-Controlled Randomised Controlled Trial. Urol. Int..

[16] Upadhyay R.K. (2016). Antidiabetic potential of plant natural products: A review. Int. J. Green Pharm..

[17] Tan X.C., Chua K.H., Ram M.R., Kuppusamy U.R. (2016). Monoterpenes: Novel insights into their biological effects and roles on glucose uptake and lipid metabolism in 3T3-L1 adipocytes. Food Chem..

[18] Jelenkovic L., Jovanovic V., Palic I., Mitic V., Radulovic M. (2014). In Vitro Screening of α-Amylase Inhibition by Selected Terpenes from Essential Oils. Trop. J. Pharm. Res..

[19] Sahin Basak S., Candan F. (2010). Chemical composition and In vitro antioxidant and antidiabetic activities of Eucalyptus Camaldulensis Dehnh. essential oil. J. Iran Chem. Soc..

[20] Enzymatic Assay of Alpha-Amylase Sigma-Aldrich. https://www.sigmaaldrich.com/technical-documents/protocols/biology/enzymatic-assay-of-a-amylase.html.

[21] Bernfeld P., Colowick S.P., Kaplan N. (1955). Amylases, α and β. Methods in Enzymology.

[22] Rubiolo P., Sgorbini B., Liberto E., Cordero C., Bicchi C. (2010). Essential oils and volatiles: Sample preparation and analysis. A review. Flavour. Fragr. J..

[23] Bicchi C., Liberto E., Matteodo M., Sgorbini B., Mondello L., Zellner B., d’Acampora Costa R., Rubiolo P. (2008). Quantitative analysis of essential oils: A complex task. Flavour. Fragr. J..

