# Supplementary material for: Bio-Guided Fractionation Driven by In Vitro α-Amylase Inhibition Assays of Essential Oils Bearing Specialized Metabolites with Potential Hypoglycemic Activity
Source: Plants (Basel). 2020 Sep 21;9(9):1242. doi: 10.3390/plants9091242 (PMC7569863; doi:10.3390/plants9091242)
Supplement: Supplementary file 1 [file plants-09-01242-s001.pdf]

Supplementary material

Bio-guided fractionation driven by in-vitro α-amylase inhibition assays of essential oils bearing specialized metabolites with potential hypoglycemic activity

Francesca Capetti[1], Cecilia Cagliero[1], Arianna Marengo[1], Carlo Bicchi[1], Patrizia Rubiolo[1], Barbara Sgorbini[1]*

[1]Dipartimento di Scienza e Tecnologia del Farmaco, Università degli Studi di Torino, via P. Giuria 9, 10125 Torino, Italy

*** Address for correspondence**

Prof. Dr. Barbara Sgorbini

Dipartimento di Scienza e Tecnologia del Farmaco,

Università degli Studi di Torino

Via Pietro Giuria 9, 10125, Turin, Italy

Phone: + 390116707135,

Fax: + 390116707162

barbara.sgorbini@unito.it

Table 1

**RTI Tab**	**RTI Exp**	**Compound**	***Artemisia vulgaris***	***Cananga odorata***	***Carum carvi***	***Cedrus atlantica***	***Chrysopogon zizanioides***	***Cinnamomum zeylanicum* (Leaf)**	***Cinnamomum zeylanicum (Bark)***	***Cinnamomum camphora***	***Cinnamomum cassia***	***Citrus × aurantium* L. *(Fruit and Peel)***	***Citrus × aurantium (Flower)***	***Citrus × aurantium (Leaf)***
**931**	930	α-Thujene	0.0	0.0	0.0	0.0	0.0	**0.1**	**0.1**	0.0	0.0	0.0	0.0	0.0
**939**	936	α-Pinene	**0.3**	**0.1**	**0.2**	0.0	0.0	**0.9**	**0.6**	0.0	**0.1**	**0.9**	0.0	**0.1**
**953**	951	Camphene	**3.7**	0.0	0.0	0.0	0.0	**0.3**	**0.2**	0.0	0.0	0.0	0.0	0.0
**961**	963	Benzaldehyde	0.0	0.0	0.0	0.0	0.0	**0.2**	**0.2**	0.0	**0.5**	0.0	0.0	0.0
**976**	976	Sabinene	**3.9**	0.0	**0.2**	0.0	0.0	0.0	**0.0**	0.0	0.0	**0.5**	0.0	**0.2**
**980**	978	β-Pinene	0.0	0.0	**0.0**	0.0	0.0	**0.3**	**0.2**	**7.6**	0.0	0.0	**7.6**	**1.0**
**991**	992	β-Myrcene	0.0	0.0	**0.7**	0.0	0.0	**0.1**	**0.0**	0.0	0.0	**3.7**	**0.8**	**1.7**
**1005**	1004	α-Phellandrene	0.0	0.0	**0.1**	0.0	0.0	**1.1**	**1.2**	**3.8**	0.0	0.0	0.0	0.0
**1001**	1005	*N*-Octanal	0.0	0.0	0.0	0.0	0.0	0.0	0.0	0.0	0.0	**0.4**	0.0	0.0
**1011**	1011	Δ-3-Carene	0.0	0.0	**0.0**	0.0	0.0	0.0	**0.1**	0.0	0.0	0.0	0.0	**0.4**
**1018**	1018	α-Terpinene	0.0	0.0	0.0	0.0	0.0	**0.1**	**0.4**	**9.6**	0.0	0.0	0.0	0.0
**1019**	1022	*p*-Methoxy toluene	0.0	**2.5**	0.0	0.0	0.0	0.0	0.0	0.0	0.0	0.0	0.0	0.0
**1026**	1028	*p*-Cymene	0.0	0.0	0.0	0.0	0.0	**0.7**	**1.6**	**14.6**	0.0	0.0	0.0	0.0
**1031**	1031	Limonene	0.0	0.0	**35.4**	0.0	0.0	**0.6**	**2.5**	**17.4**	0.0	**90.2**	**11.4**	**0.9**
**1033**	1033	1,8-Cineole	**0.7**	**0.1**	0.0	0.0	0.0	**0.1**	**0.2**	**44.1**	0.0	0.0	0.0	0.0
**1040**	1041	*cis*-β-Ocimene	0.0	0.0	0.0	0.0	0.0	0.0	0.0	0.0	0.0	0.0	**1.2**	**0.6**
**1041**	1045	Salicylaldehyde	0.0	0.0	0.0	0.0	0.0	0.0	0.0	0.0	**0.2**	0.0	0.0	0.0
**1050**	1051	*trans*-β-Ocimene	0.0	0.0	0.0	0.0	0.0	0.0	**0.1**	0.0	0.0	0.0	**2.6**	**1.7**
**1062**	1061	γ-Terpinene	**0.4**	0.0	0.0	0.0	0.0	0.0	0.0	**0.5**	0.0	0.0	0.0	0.0
**1088**	1089	α-Terpinolene	0.0	0.0	0.0	0.0	0.0	0.0	**0.2**	0.0	0.0	0.0	0.0	**0.3**
**1091**	1094	Methyl benzoate	0.0	**1.2**	0.0	0.0	0.0	0.0	0.0	0.0	0.0	0.0	0.0	0.0
**1098**	1100	Linalool	0.0	**4.2**	0.0	0.0	0.0	**1.8**	**4.4**	0.0	0.0	0.0	**28.5**	**24.4**
**1102**	1107	α-Thujone	**47.4**	0.0	0.0	0.0	0.0	0.0	0.0	0.0	0.0	0.0	0.0	0.0
**1110**	1115	Phenethyl alcohol	0.0	0.0	0.0	0.0	0.0	0.0	0.0	0.0	**0.2**	0.0	0.0	0.0
**1114**	1118	β-Thujone	**7.8**	0.0	0.0	0.0	0.0	0.0	0.0	0.0	0.0	0.0	0.0	0.0
**1143**	1147	Camphor	**30.0**	0.0	0.0	0.0	0.0	0.0	0.0	0.0	0.0	0.0	0.0	0.0
**1165**	1165	Borneol	**1.0**	0.0	0.0	0.0	0.0	0.0	0.0	0.0	0.0	0.0	0.0	0.0
**1164**	1169	Benzyl acetate	0.0	**0.9**	0.0	0.0	0.0	0.0	0.0	0.0	0.0	0.0	0.0	0.0
**1170**	1174	Ethyl benzoate	0.0	**0.2**	0.0	0.0	0.0	0.0	0.0	0.0	0.0	0.0	0.0	0.0
**1177**	1178	4-Terpineol	**1.0**	0.0	0.0	0.0	0.0	0.0	0.0	0.0	0.0	0.0	0.0	0.0
**1189**	1191	α-Terpineol	0.0	0.0	0.0	0.0	0.0	**0.2**	**0.5**	0.0	0.0	0.0	**1.1**	**5.6**

RTI Tab	RTI Exp	Compound	*Artemisia vulgaris*	*Cananga odorata*	*Carum carvi*	*Cedrus atlantica*	*Chrysopogon zizanioides*	*Cinnamomum zeylanicum* (Leaf)	*Cinnamomum zeylanicum (Bark)*	*Cinnamomum camphora*	*Cinnamomum cassia*	*Citrus × aurantium L. (Fruit and Peel)*	*Citrus × aurantium (Flower)*	*Citrus × aurantium (Leaf)*
**1193**	1195	*cis*-Dihydroxy carvone	0.0	0.0	**0.6**	0.0	0.0	0.0	0.0	0.0	0.0	0.0	0.0	0.0
**1200**	1202	*trans*-Dihydroxy carvone	0.0	0.0	**0.2**	0.0	0.0	0.0	0.0	0.0	0.0	0.0	0.0	0.0
**1204**	1207	*N*-Decanal	0.0	0.0	0.0	0.0	0.0	0.0	0.0	0.0	0.0	**0.1**	0.0	0.0
**1228**	1232	Nerol	0.0	0.0	0.0	0.0	0.0	0.0	0.0	0.0	0.0	0.0	0.0	**0.4**
**1252**	1244	*p*-Anisaldehyde	0.0	0.0	0.0	0.0	0.0	0.0	0.0	0.0	**0.1**	0.0	0.0	0.0
**1242**	1253	Carvone	0.0	0.0	**59.6**	0.0	0.0	0.0	0.0	0.0	0.0	0.0	0.0	0.0
**1255**	1258	Geraniol	0.0	**0.7**	0.0	0.0	0.0	0.0	0.0	0.0	0.0	0.0	0.0	0.0
**1257**	1264	Linalyl acetate	0.0	0.0	0.0	0.0	0.0	0.0	0.0	0.0	0.0	**1.6**	**41.4**	**56.8**
**1266**	1269	*trans*-Cinnamaldehyde	0.0	0.0	0.0	0.0	0.0	**0.6**	**68.0**	0.0	**80.4**	0.0	0.0	0.0
**1285**	1288	Safrole	0.0	0.0	0.0	0.0	0.0	**0.8**	0.0	0.0	0.0	0.0	0.0	0.0
**1300**	1305	Cinnamyl alcohol	0.0	0.0	0.0	0.0	0.0	0.0	0.0	0.0	**0.1**	0.0	0.0	0.0
**1356**	1360	Eugenol	0.0	**0.6**	0.0	0.0	0.0	**82.4**	**5.1**	0.0	0.0	0.0	0.0	0.0
**1365**	1369	Neryl acetate	0.0	0.0	0.0	0.0	0.0	0.0	0.0	0.0	0.0	0.0	**0.5**	**1.8**
**1372**	1377	α-Copaene	0.0	**1.5**	0.0	0.0	0.0	**0.7**	**0.8**	0.0	**0.3**	0.0	0.0	0.0
**1383**	1386	Geranyl acetate	0.0	**4.6**	0.0	0.0	0.0	0.0	0.0	0.0	0.0	**0.3**	**1.2**	**3.4**
**1391**	1393	β-Elemene	0.0	**0.2**	0.0	0.0	0.0	0.0	0.0	0.0	0.0	0.0	0.0	0.0
**1402**	1408	Methyl N-methylanthranilate	0.0	0.0	0.0	0.0	0.0	0.0	0.0	0.0	0.0	0.0	**0.7**	0.0
**1418**	1419	*trans*-β-Caryophyllene	0.0	**20.5**	**0.1**	0.0	0.0	**3.0**	**6.5**	0.0	0.0	**0.2**	**0.1**	**0.5**
**1429**	1434	Cumarin	0.0	0.0	0.0	0.0	0.0	0.0	0.0	0.0	**1.9**	0.0	0.0	0.0
**-**	1441	khusimene	0.0	0.0	0.0	0.0	**3.0**	0.0	0.0	0.0	0.0	0.0	0.0	0.0
**1451**	1448	α-Himachalene	0.0	0.0	0.0	**16.8**	0.0	0.0	0.0	0.0	0.0	0.0	0.0	0.0
**1443**	1449	*trans*-Cinnamyl acetate	0.0	**0.6**	0.0	0.0	0.0	**0.8**	**1.5**	0.0	**2.6**	0.0	0.0	0.0
**1454**	1455	α-Humulene	0.0	**4.9**	0.0	0.0	0.0	**0.4**	**1.1**	0.0	0.0	0.0	0.0	0.0
**-**	1477	γ-Himachalene	0.0	0.0	0.0	**12.1**	0.0	0.0	0.0	0.0	0.0	0.0	0.0	0.0
**1480**	1480	Germacrene D	**0.4**	**15.6**	0.0	0.0	0.0	0.0	0.0	0.0	0.0	0.0	0.0	0.0
**1499**	1499	β-Himachalene	0.0	0.0	0.0	**48.8**	0.0	0.0	0.0	0.0	0.0	0.0	0.0	0.0
**-**	1509	γ-vetivenene	0.0	0.0	0.0	0.0	**2.9**	0.0	0.0	0.0	0.0	0.0	0.0	0.0
**1509**	1510	β-Bisabolene	0.0	0.0	0.0	0.0	0.0	0.0	0.0	0.0	**0.1**	0.0	0.0	0.0
**1508**	1511	*trans*-α-Farnesene	0.0	**7.9**	0.0	0.0	0.0	0.0	0.0	0.0	0.0	0.0	0.0	0.0
**-**	1523	β-vetivenene	0.0	0.0	0.0	0.0	**3.7**	0.0	0.0	0.0	0.0	0.0	0.0	0.0

RTI Tab	RTI Exp	Compound	*Artemisia vulgaris*	*Cananga odorata*	*Carum carvi*	*Cedrus atlantica*	*Chrysopogon zizanioides*	*Cinnamomum zeylanicum* (Leaf)	*Cinnamomum zeylanicum (Bark)*	*Cinnamomum camphora*	*Cinnamomum cassia*	*Citrus × aurantium* L. *(Fruit and Peel)*	*Citrus × aurantium (Flower)*	*Citrus × aurantium (Leaf)*
**1524**	1524	δ-Cadinene	0.0	**5.4**	0.0	**4.1**	0.0	0.0	0.0	0.0	**0.1**	0.0	0.0	0.0
**1524**	1528	*trans-o*-methoxy Cinnamaldehyde	0.0	0.0	0.0	0.0	0.0	**0.1**	**0.3**	0.0	**11.2**	0.0	0.0	0.0
**1534**	1537	*cis*-Nerolidol	0.0	0.0	0.0	0.0	0.0	0.0	0.0	0.0	0.0	0.0	**0.8**	0.0
**1538**	1538	α-Cadinene	0.0	**0.3**	0.0	0.0	0.0	0.0	0.0	0.0	0.0	0.0	0.0	0.0
**1581**	1578	Spathulenol	0.0	0.0	**0.2**	0.0	0.0	**0.2**	**0.7**	0.0	0.0	0.0	0.0	0.0
**1701**	1705	*trans*-γ-Atlantone	0.0	0.0	0.0	**2.6**	0.0	0.0	0.0	0.0	0.0	0.0	0.0	0.0
**-**	1723	khusinol (zizanol)	0.0	0.0	0.0	0.0	**4.5**	0.0	0.0	0.0	0.0	0.0	0.0	0.0
**1722**	1724	*trans-trans*-Farnesolo	0.0	**1.6**	0.0	0.0	0.0	0.0	0.0	0.0	0.0	0.0	0.0	0.0
**1762**	1766	Benzyl benzoate	0.0	**8.9**	0.0	0.0	0.0	**2.5**	**1.0**	0.0	0.0	0.0	0.0	0.0
**1773**	1776	trans-α-Atlantone	0.0	0.0	0.0	**0.7**	0.0	0.0	0.0	0.0	0.0	0.0	0.0	0.0
**-**	1798	β-vetivone	0.0	0.0	0.0	0.0	**1.4**	0.0	0.0	0.0	0.0	0.0	0.0	0.0
**-**	1819	α-vetivone	0.0	0.0	0.0	0.0	**1.7**	0.0	0.0	0.0	0.0	0.0	0.0	0.0
**1843**	1845	*trans-trans*-Farnesyl acetate	0.0	**1.8**	0.0	0.0	0.0	0.0	0.0	0.0	0.0	0.0	0.0	0.0
**1863**	1868	Benzyl salicilate	0.0	**2.1**	0.0	0.0	0.0	0.0	0.0	0.0	0.0	0.0	0.0	0.0

Table 2

RTI Tab	RTI Exp	Compound	*Citrus bergamia*	*Citrus limon*	*Citrus medica*	*Citrus nobilis*	*Citrus paradisi*	*Citrus sinensis*	*Corymbia citriodora*	*Cupressus sempervirens*	*Cymbopogon martini*	*Cymbopogon nardus*
**931**	930	α-Thujene	0.0	**0.3**	**0.1**	**0.2**	0.0	0.0	0.0	0.0	0.0	0.0
**939**	936	α-Pinene	**1.0**	**1.5**	**1.7**	**1.9**	**0.9**	**0.5**	**0.3**	**46.7**	0.0	0.0
**953**	951	Camphene	0.0	0.0	0.0	0.0	0.0	0.0	0.0	**0.6**	0.0	0.0
**976**	976	Sabinene	**0.9**	**1.4**	**0.7**	**0.4**	**0.6**	**0.4**	0.0	**0.9**	0.0	0.0
**980**	978	β-Pinene	**6.6**	**11.6**	**9.1**	**1.1**	0.0	**0.0**	**0.6**	**1.3**	0.0	0.0
**991**	992	β-Myrcene	**0.9**	**1.5**	**1.5**	**1.0**	**3.0**	**1.7**	0.0	**2.3**	0.0	**0.7**
**1005**	1004	α-Phellandrene	0.0	0.0	**0.1**	**0.2**	0.0	0.0	0.0	0.0	0.0	0.0
**1001**	1005	*N*-Octanal	0.0	0.0	0.0	0.0	**0.4**	**0.1**	0.0	0.0	0.0	0.0
**1011**	1011	Δ-3-Carene	0.0	0.0	0.0	0.0	0.0	**0.1**	0.0	**25.3**	0.0	0.0
**1018**	1018	α-Terpinene	**0.2**	0.0	0.0	**0.6**	0.0	0.0	0.0	**0.5**	0.0	0.0
**1026**	1028	*p*-Cymene	**0.4**	**0.2**	**0.3**	**0.7**	0.0	0.0	0.0	0.0	0.0	0.0
**1031**	1031	Limonene	**32.3**	**71.9**	**54.9**	**75.6**	**92.7**	**96.7**	**0.1**	**4.0**	0.0	**7.2**
**1033**	1033	1,8-Cineole	0.0	0.0	0.0	0.0	0.0	0.0	**0.8**	0.0	0.0	0.0
**1040**	1041	*cis*-β-Ocimene	0.0	0.0	0.0	0.0	0.0	0.0	0.0	0.0	**1.1**	0.0
**1050**	1051	*trans*-β-Ocimene	**0.1**	0.0	**0.1**	0.0	0.0	0.0	0.0	0.0	**1.7**	0.0
**1062**	1061	γ-Terpinene	**7.8**	**7.9**	**3.2**	**14.5**	0.0	**0.0**	0.0	**0.8**	0.0	0.0
**1088**	1089	α-Terpinolene	**0.5**	**0.3**	**0.1**	**0.6**	0.0	**0.0**	0.0	**4.2**	0.0	0.0
**1098**	1100	Linalool	**11.2**	0.0	**4.6**	0.0	0.0	**0.2**	**0.3**	0.0	**2.1**	**0.6**
**1145**	1148	Neoisopulegol	0.0	0.0	0.0	0.0	0.0	0.0	**7.1**	0.0	0.0	**1.5**
**1153**	1158	Citronellal	0.0	0.0	0.0	0.0	0.0	0.0	**81.0**	0.0	0.0	**37.9**
**1177**	1178	4-Terpineol	0.0	0.0	0.0	0.0	0.0	0.0	0.0	**1.5**	0.0	0.0
**1189**	1191	α-Terpineol	0.0	**0.1**	**0.0**	0.0	0.0	0.0	0.0	0.0	0.0	0.0
**1204**	1207	*N*-Decanal	0.0	0.0	0.0	0.0	**0.4**	**0.1**	0.0	0.0	0.0	0.0
**1228**	1232	Citronellol	0.0	0.0	0.0	0.0	0.0	0.0	**6.0**	0.0	0.0	**12.5**

RTI Tab	RTI Exp	Compound	*Citrus bergamia*	*Citrus limon*	*Citrus medica*	*Citrus nobilis*	*Citrus paradisi*	*Citrus sinensis*	*Corymbia citriodora*	*Cupressus sempervirens*	*Cymbopogon martini*	*Cymbopogon nardus*
**1240**	1244	Neral	**0.3**	**0.8**	**2.9**	0.0	0.0	**0.0**	0.0	0.0	0.0	**2.0**
**1255**	1258	Geraniol	0.0	0.0	0.0	0.0	0.0	0.0	0.0	0.0	**84.5**	**19.5**
**1257**	1264	Linalyl acetate	**34.1**	0.0	**14.5**	0.0	0.0	0.0	0.0	0.0	0.0	0.0
**1270**	1273	Geranial	**0.5**	**1.3**	**4.6**	0.0	0.0	0.0	0.0	0.0	**0.6**	**2.2**
**1350**	1343	α-Terpinyl acetate	0.0	0.0	0.0	0.0	0.0	0.0	0.0	**3.3**	0.0	0.0
**1354**	1356	Citronellyl acetate	0.0	0.0	0.0	0.0	0.0	0.0	**1.5**	0.0	0.0	**3.2**
**1365**	1369	Neryl acetate	**0.6**	**0.3**	**0.4**	0.0	0.0	0.0	0.0	0.0	0.0	0.0
**1383**	1386	Geranyl acetate	**0.5**	**0.3**	**0.2**	0.0	0.0	0.0	0.0	0.0	**7.8**	**4.4**
**1391**	1393	β-Elemene	0.0	0.0	0.0	0.0	0.0	0.0	0.0	0.0	0.0	**0.6**
**1402**	1408	Methyl N-methylanthranilate	0.0	0.0	0.0	**0.5**	0.0	0.0	0.0	0.0	0.0	0.0
**1409**	1411	α-Cedrene	0.0	0.0	0.0	0.0	0.0	0.0	0.0	**0.7**	0.0	0.0
**1418**	1419	*trans*-β-Caryophyllene	**0.2**	**0.1**	**0.1**	0.0	**0.3**	0.0	**0.8**	0.0	**1.2**	0.0
**1480**	1480	Germacrene D	0.0	0.0	0.0	0.0	0.0	0.0	0.0	0.0	0.0	**0.9**
**1491**	1493	Valencene	0.0	0.0	0.0	0.0	0.0	**0.1**	0.0	0.0	0.0	0.0
**1499**	1498	α-Muurolene	0.0	0.0	0.0	0.0	0.0	0.0	0.0	0.0	0.0	**0.3**
**1509**	1510	β-Bisabolene	**0.3**	0.0	0.0	0.0	0.0	0.0	0.0	0.0	0.0	0.0
**1513**	1514	*trans*-γ-Cadinene	0.0	0.0	0.0	0.0	0.0	0.0	0.0	0.0	0.0	**0.5**
**1524**	1524	δ-Cadinene	0.0	0.0	0.0	0.0	0.0	0.0	0.0	0.0	0.0	**1.1**
**1549**	1551	Elemol	0.0	0.0	0.0	0.0	0.0	0.0	0.0	0.0	0.0	**1.6**
**1574**	1572	*trans*-3-Hexen-1-yl benzoate	0.0	0.0	0.0	0.0	0.0	0.0	0.0	0.0	0.0	**0.4**
**1596**	1599	α-Cedrol	0.0	0.0	0.0	0.0	0.0	0.0	0.0	**2.9**	0.0	0.0
**1653**	1656	α-Cadinol	0.0	0.0	0.0	0.0	0.0	0.0	0.0	0.0	0.0	**0.5**

Table 3

RTI Tab	RTI Exp	Compound	*Cymbopogon schoenanthus*	*Elettaria cardamomum*	*Eucalyptus globulus*	*Eucalyptus radiata*	*Foeniculum Vulgare*	*Gaultheria procumbens*	*Hyssopus officinalis*	*Jasminum officinale*	*Juniperus communis*	*Juniperus virginiana*
**931**	930	α-Thujene	0.0	**0.2**	0.0	**0.2**	0.0	0.0	**0.3**	0.0	**1.3**	0.0
**939**	936	α-Pinene	**0.2**	**1.3**	**2.2**	**2.7**	**1.3**	0.0	**7.1**	0.0	**35.9**	0.0
**953**	951	Camphene	**0.6**	0.0	0.0	0.0	**0.1**	0.0	**0.3**	0.0	**0.5**	0.0
**976**	976	Sabinene	0.0	**2.9**	0.0	**1.0**	**0.1**	0.0	**5.8**	0.0	**8.4**	0.0
**980**	978	β-Pinene	0.0	**0.3**	**0.3**	**0.7**	**0.5**	0.0	**5.6**	0.0	**5.4**	0.0
**985**	989	6-Methyl-5-hepten-2-one	**0.8**	0.0	0.0	0.0	0.0	0.0	0.0	0.0	0.0	0.0
**991**	992	β-Myrcene	0.0	**1.1**	**0.4**	**0.5**	**1.3**	0.0	**0.9**	0.0	**14.2**	0.0
**1005**	1004	α-Phellandrene	0.0	0.0	**0.6**	0.0	**4.7**	0.0	0.0	0.0	**0.5**	0.0
**1011**	1011	Δ-3-Carene	0.0	0.0	0.0	0.0	**0.1**	0.0	0.0	0.0	0.0	0.0
**1018**	1018	α-Terpinene	0.0	**0.1**	0.0	**4.4**	0.0	0.0	0.0	0.0	**0.6**	0.0
**1026**	1028	*p*-Cymene	0.0	**0.3**	**3.1**	**0.8**	**0.5**	0.0	**3.0**	0.0	**2.7**	0.0
**1031**	1031	Limonene	**2.7**	**2.2**	**6.8**	**4.3**	**3.6**	0.0	**1.8**	0.0	**8.0**	0.0
**1033**	1033	1,8-Cineole	0.0	**34.7**	**82.1**	**75.1**	**0.1**	0.0	**39.2**	0.0	0.0	0.0
**1040**	1041	*cis*-β-Ocimene	0.0	0.0	0.0	0.0	**0.4**	0.0	**0.2**	0.0	0.0	0.0
**1050**	1051	*trans*-β-Ocimene	0.0	0.0	0.0	0.0	**0.1**	0.0	0.0	0.0	0.0	0.0
**1062**	1061	γ-Terpinene	0.0	**0.3**	**3.3**	0.0	**0.2**	0.0	**2.9**	0.0	**2.4**	0.0
**1068**	1070	*cis*-Sabinene hydrate	0.0	**0.1**	0.0	0.0	0.0	0.0	0.0	0.0	0.0	0.0
**1087**	1089	Fenchone	0.0	0.0	0.0	0.0	**3.4**	0.0	0.0	0.0	0.0	0.0
**1088**	1089	α-Terpinolene	0.0	**0.1**	0.0	0.0	0.0	0.0	0.0	0.0	**1.7**	0.0
**1097**	1098	*trans*-Sabinene hydrate	0.0	**0.1**	0.0	0.0	0.0	0.0	0.0	0.0	0.0	0.0
**1098**	1100	Linalool	**0.9**	**2.7**	0.0	**0.3**	0.0	0.0	**2.9**	**9.4**	0.0	0.0
**1143**	1147	Camphor	0.0	0.0	0.0	0.0	**0.1**	0.0	0.0	0.0	0.0	0.0
**1163**	1164	Pinocamphone	0.0	0.0	0.0	0.0	0.0	0.0	**2.4**	0.0	0.0	0.0
**1164**	1169	Benzyl acetate	0.0	0.0	0.0	0.0	0.0	0.0	0.0	**30.0**	0.0	0.0
**1173**	1177	Isopinocamphone	0.0	0.0	0.0	0.0	0.0	0.0	**6.1**	0.0	0.0	0.0
**1177**	1178	4-Terpineol	0.0	**0.7**	**0.1**	**1.0**	0.0	0.0	**2.0**	0.0	**3.5**	0.0
**1188**	1188	Neoisomenthol	0.0	0.0	0.0	0.0	0.0	0.0	0.0	0.0	0.0	0.0
**1189**	1191	α-Terpineol	0.0	**1.3**	**0.1**	**7.6**	0.0	0.0	0.0	0.0	**0.4**	0.0
**1190**	1194	Methyl salicylate	0.0	0.0	0.0	0.0	0.0	**99.7**	0.0	0.0	0.0	0.0
**1193**	1195	*cis*-Dihydroxy carvone	0.0	0.0	0.0	0.0	0.0	0.0	0.0	0.0	0.0	0.0

RTI Tab	RTI Exp	Compound	*Cymbopogon schoenanthus*	*Elettaria cardamomum*	*Eucalyptus globulus*	*Eucalyptus radiata*	*Foeniculum Vulgare*	*Gaultheria procumbens*	*Hyssopus officinalis*	*Jasminum officinale*	*Juniperus communis*	*Juniperus virginiana*
**1195**	1199	Estragole	0.0	0.0	0.0	0.0	**1.0**	0.0	0.0	0.0	0.0	0.0
**1240**	1244	Neral	**32.5**	0.0	0.0	**0.4**	0.0	0.0	0.0	0.0	0.0	0.0
**1255**	1258	Geraniol	**7.3**	0.0	0.0	0.0	0.0	0.0	0.0	0.0	0.0	0.0
**1257**	1264	Linalyl acetate	0.0	**6.0**	0.0	0.0	0.0	0.0	0.0	0.0	0.0	0.0
**1266**	1269	*trans*-Cinnamaldehyde	0.0	0.0	0.0	0.0	0.0	0.0	0.0	0.0	0.0	0.0
**1270**	1273	Geranial	**38.2**	**0.2**	0.0	**0.5**	0.0	0.0	0.0	0.0	0.0	0.0
**1285**	1287	Bornyl acetate	0.0	0.0	0.0	0.0	0.0	0.0	0.0	0.0	**0.2**	0.0
**1288**	1293	Indole	0.0	0.0	0.0	0.0	0.0	0.0	0.0	**1.3**	0.0	0.0
**1283**	1301	*trans*-Anethol	0.0	0.0	0.0	0.0	**82.0**	0.0	0.0	0.0	0.0	0.0
**1350**	1343	α-Terpinyl acetate	0.0	**43.8**	0.0	0.0	0.0	0.0	0.0	0.0	0.0	0.0
**1351**	1351	α-Cubebene	0.0	0.0	0.0	0.0	0.0	0.0	0.0	0.0	**0.6**	0.0
**1356**	1360	Eugenol	0.0	0.0	0.0	0.0	0.0	0.0	0.0	**2.9**	0.0	0.0
**1372**	1377	α-Copaene	0.0	0.0	0.0	0.0	0.0	0.0	0.0	0.0	**0.3**	0.0
**1383**	1386	Geranyl acetate	**4.2**	**0.2**	0.0	0.0	0.0	0.0	0.0	0.0	0.0	0.0
**1384**	1392	β-Bourbonene	0.0	0.0	0.0	0.0	0.0	0.0	**0.2**	0.0	0.0	0.0
**1391**	1393	β-Elemene	0.0	0.0	0.0	0.0	0.0	0.0	0.0	0.0	**0.9**	0.0
**1388**	1399	*trans*-Jasmone	0.0	0.0	0.0	0.0	0.0	0.0	0.0	**2.2**	0.0	0.0
**1401**	1407	Methyl eugenol	0.0	0.0	0.0	0.0	0.0	0.0	**1.8**	0.0	0.0	0.0
**1409**	1412	α-Himalachene	0.0	0.0	0.0	0.0	0.0	0.0	0.0	0.0	0.0	**16.0**
**1418**	1419	*trans*-β-Caryophyllene	**2.9**	0.0	0.0	0.0	0.0	0.0	**0.4**	0.0	**4.4**	0.0
**1429**	1433	γ-Himalachene	0.0	0.0	0.0	0.0	0.0	0.0	0.0	0.0	0.0	**10.0**
**1433**	1435	γ-Elemene	0.0	0.0	0.0	0.0	0.0	0.0	0.0	0.0	**0.2**	0.0
**1447**	1450	*trans*-Isoeugenol	0.0	0.0	0.0	0.0	0.0	0.0	0.0	**0.2**	0.0	0.0
**1454**	1455	α-Humulene	0.0	0.0	0.0	0.0	0.0	0.0	0.0	0.0	**1.2**	0.0
**1477**	1477	γ-Muurolene	0.0	0.0	0.0	0.0	0.0	0.0	0.0	0.0	**0.4**	0.0
**1480**	1480	Germacrene D	0.0	0.0	0.0	0.0	0.0	0.0	**0.3**	0.0	**1.6**	0.0
**1485**	1485	β-Selinene	0.0	**0.3**	0.0	0.0	0.0	0.0	0.0	0.0	0.0	0.0
**1485**	1493	*trans*-Jasmolactone	0.0	0.0	0.0	0.0	0.0	0.0	0.0	**0.7**	0.0	0.0
**1494**	1495	Bicyclogermacrene	0.0	0.0	0.0	0.0	0.0	0.0	**0.3**	0.0	0.0	0.0
**1499**	1499	β-Himachalene	0.0	0.0	0.0	0.0	0.0	0.0	0.0	0.0	0.0	**50.8**
**1500**	1499	α-Chamigrene	0.0	0.0	0.0	0.0	0.0	0.0	0.0	0.0	0.0	**2.0**
**1513**	1514	*trans*-γ-Cadinene	0.0	0.0	0.0	0.0	0.0	0.0	0.0	0.0	0.0	**1.1**

RTI Tab	RTI Exp	Compound	*Cymbopogon schoenanthus*	*Elettaria cardamomum*	*Eucalyptus globulus*	*Eucalyptus radiata*	*Foeniculum Vulgare*	*Gaultheria procumbens*	*Hyssopus officinalis*	*Jasminum officinale*	*Juniperus communis*	*Juniperus virginiana*
**1524**	1524	δ-Cadinene	0.0	0.0	0.0	0.0	0.0	0.0	0.0	0.0	**0.7**	**2.5**
**1570**	1556	Menthyl acetate	0.0	0.0	0.0	0.0	0.0	0.0	0.0	**1.2**	0.0	0.0
**1564**	1568	trans-Nerolidol	0.0	**0.2**	0.0	0.0	0.0	0.0	0.0	0.0	0.0	0.0
**1576**	1577	Germacrene D-4-ol	0.0	0.0	0.0	0.0	0.0	0.0	**0.2**	0.0	0.0	0.0
**1581**	1578	Spathulenol	**0.6**	0.0	0.0	0.0	0.0	0.0	0.0	0.0	**0.3**	0.0
**1654**	1651	*cis*-Methyl-dihydrojasmoato	0.0	0.0	0.0	0.0	0.0	0.0	0.0	**0.5**	0.0	0.0
**1762**	1766	Benzyl benzoate	0.0	0.0	0.0	0.0	0.0	0.0	0.0	**2.2**	0.0	0.0
**1949**	1949	Fitol	0.0	0.0	0.0	0.0	0.0	0.0	0.0	**6.3**	0.0	0.0

Table 4

RTI Tab	RTI Exp	Compound	*Laurus nobilis*	*Lavandula angustifolia* × *L. latifolia*	*Lavandula angustifolia*	*Matricaria chamomilla*	*Melaleuca alternifolia*	*Melaleuca viridiflora*	*Melissa officinalis*	*Mentha × piperita (Leaf)*	*Mentha × piperita (Leaf/Twig)*	*Mentha arvensis*
**926**	924	Tricyclene	0.0	0.0	0.0	0.0	0.0	**0.1**	0.0	0.0	0.0	0.0
**931**	930	α-Thujene	**0.2**	0.0	0.0	0.0	**0.8**	0.0	0.0	0.0	0.0	0.0
**939**	936	α-Pinene	**6.4**	**0.7**	0.0	0.0	**2.4**	**6.2**	**0.7**	**0.1**	0.0	0.0
**953**	951	Camphene	**0.2**	0.0	0.0	0.0	0.0	0.0	0.0	0.0	0.0	0.0
**961**	963	Benzaldehyde	0.0	0.0	0.0	0.0	0.0	**0.2**	0.0	0.0	0.0	0.0
**976**	976	Sabinene	**5.1**	0.0	0.0	0.0	0.0	0.0	**0.5**	0.0	**0.0**	0.0
**980**	978	β-Pinene	**3.8**	**0.6**	0.0	0.0	**0.8**	**1.5**	**0.9**	**0.1**	**0.1**	**1.5**
**986**	989	3-Octanone	0.0	0.0	**0.8**	0.0	0.0	0.0	0.0	0.0	0.0	0.0
**991**	992	β-Myrcene	**0.3**	**0.6**	0.0	0.0	**0.8**	**0.7**	**0.8**	0.0	0.0	0.0
**1005**	1004	α-Phellandrene	**0.1**	0.0	0.0	0.0	**0.5**	0.0	0.0	0.0	0.0	0.0
**1018**	1018	α-Terpinene	**0.2**	0.0	0.0	0.0	**9.4**	**0.2**	0.0	0.0	0.0	0.0
**1019**	1022	*p*-Methoxy toluene	0.0	0.0	0.0	0.0	0.0	0.0	0.0	0.0	0.0	0.0
**1026**	1028	*p*-Cymene	**1.9**	0.0	0.0	0.0	**1.9**	**0.7**	0.0	0.0	**0.1**	0.0
**1031**	1031	Limonene	0.0	**0.9**	**0.7**	0.0	**0.8**	**9.5**	**6.4**	**1.0**	**0.6**	**4.5**
**1033**	1033	1,8-Cineole	**65.4**	**7.6**	**2.3**	0.0	**3.2**	**64.9**	**1.6**	**1.5**	**1.5**	**0.5**
**1040**	1041	*cis*-β-Ocimene	0.0	**1.0**	**2.6**	0.0	0.0	0.0	0.0	0.0	0.0	0.0
**1050**	1051	*trans*-β-Ocimene	0.0	0.0	**2.0**	**0.3**	0.0	0.0	0.0	0.0	0.0	0.0
**1062**	1061	γ-Terpinene	**2.4**	0.0	0.0	**0.2**	**21.1**	**1.1**	0.0	0.0	0.0	0.0
**1062**	1065	Artemisia Ketone	0.0	0.0	0.0	**0.4**	0.0	0.0	0.0	0.0	0.0	0.0
**1068**	1070	*cis*-Sabinene hydrate	**0.2**	0.0	0.0	0.0	0.0	0.0	0.0	0.0	0.0	0.0
**1083**	1086	Artemisyl alcohol	0.0	0.0	0.0	**0.1**	0.0	0.0	0.0	0.0	0.0	0.0
**1088**	1089	α-Terpinolene	**0.1**	0.0	0.0	0.0	**3.2**	**0.5**	0.0	0.0	0.0	0.0
**1097**	1098	*trans*-Sabinene hydrate	**0.1**	0.0	0.0	0.0	0.0	0.0	0.0	0.0	0.0	0.0
**1098**	1100	Linalool	**2.2**	**27.1**	**27.9**	0.0	0.0	**0.0**	**1.2**	0.0	0.0	0.0
**1110**	1116	1-Octen-3-yl acetate	0.0	0.0	**0.7**	0.0	0.0	0.0	0.0	0.0	0.0	0.0
**1146**	1146	Isopulegol	0.0	0.0	0.0	0.0	0.0	0.0	0.0	**1.2**	0.0	**2.2**
**1143**	1147	Camphor	0.0	**9.4**	**2.0**	0.0	0.0	0.0	0.0	0.0	0.0	0.0
**1154**	1157	Menthone	0.0	0.0	0.0	0.0	0.0	0.0	0.0	**24.9**	**23.0**	**19.5**
**1153**	1158	Citronellal	0.0	0.0	0.0	0.0	0.0	0.0	**0.4**	0.0	0.0	0.0

RTI Tab	RTI Exp	Compound	*Laurus nobilis*	*Lavandula angustifolia* × *L. latifolia*	*Lavandula angustifolia*	*Matricaria chamomilla*	*Melaleuca alternifolia*	*Melaleuca viridiflora*	*Melissa officinalis*	*Mentha × piperita (Leaf)*	*Mentha × piperita (Leaf/Twig)*	*Mentha arvensis*
**1165**	1165	Borneol	0.0	**3.4**	0.0	0.0	0.0	0.0	0.0	0.0	0.0	0.0
**1163**	1165	Neomenthol	0.0	0.0	0.0	0.0	0.0	0.0	0.0	**2.0**	**4.3**	**5.2**
**1164**	1167	Isomenthone	0.0	0.0	0.0	0.0	0.0	0.0	0.0	**13.3**	**10.0**	**8.0**
**1166**	1171	Lavandulol	0.0	0.0	**0.9**	0.0	0.0	0.0	0.0	0.0	0.0	0.0
**1173**	1174	Menthol	0.0	0.0	0.0	0.0	0.0	0.0	0.0	**48.2**	**52.0**	**40.2**
**1177**	1178	4-Terpineol	**1.7**	**3.3**	**3.7**	0.0	**44.1**	**0.5**	0.0	0.0	**0.2**	0.0
**1188**	1188	Neoisomenthol	0.0	0.0	0.0	0.0	0.0	0.0	0.0	0.0	**0.4**	0.0
**1189**	1191	α-Terpineol	**0.8**	**1.2**	**0.2**	0.0	**2.6**	**4.2**	**0.3**	0.0	0.0	0.0
**1240**	1244	Neral	0.0	0.0	0.0	0.0	0.0	0.0	**18.9**	0.0	0.0	0.0
**1252**	1254	Piperitone	0.0	0.0	0.0	0.0	0.0	0.0	0.0	**0.7**	**0.1**	**1.9**
**1255**	1258	Geraniol	0.0	0.0	0.0	0.0	0.0	0.0	**1.5**	0.0	0.0	0.0
**1257**	1264	Linalyl acetate	0.0	**35.6**	**34.7**	0.0	0.0	0.0	0.0	0.0	0.0	0.0
**1270**	1273	Geranial	0.0	0.0	0.0	0.0	0.0	0.0	**24.6**	0.0	0.0	0.0
**1289**	1293	Lavandulyl acetate	0.0	**2.8**	**3.7**	0.0	0.0	0.0	0.0	0.0	0.0	0.0
**1294**	1294	Menthyl acetate	0.0	0.0	0.0	0.0	0.0	0.0	0.0	**6.4**	**4.6**	**7.4**
**1350**	1343	α-Terpinyl acetate	**8.3**	0.0	0.0	0.0	0.0	**0.7**	0.0	0.0	0.0	0.0
**1356**	1360	Eugenol	**0.6**	0.0	0.0	0.0	0.0	0.0	0.0	0.0	0.0	0.0
**1372**	1377	α-Copaene	0.0	0.0	0.0	0.0	0.0	0.0	**0.7**	0.0	0.0	0.0
**1383**	1386	Geranyl acetate	0.0	0.0	0.0	0.0	0.0	0.0	**1.2**	0.0	0.0	0.0
**1391**	1393	β-Elemene	0.0	0.0	0.0	**0.1**	0.0	0.0	0.0	0.0	0.0	0.0
**1401**	1407	Methyl eugenol	**1.4**	0.0	0.0	0.0	0.0	0.0	0.0	0.0	0.0	0.0
**1418**	1419	*trans*-β-Caryophyllene	**0.1**	**2.5**	**4.2**	0.0	0.0	**0.9**	**31.0**	0.0	**0.1**	0.0
**1439**	1439	Aromadendrene	0.0	0.0	0.0	0.0	**0.7**	0.0	0.0	0.0	0.0	0.0
**1454**	1455	α-Humulene	0.0	0.0	0.0	0.0	0.0	**0.2**	**3.5**	0.0	0.0	0.0
**1458**	1460	*trans*-β-Farnesene	0.0	**1.6**	**2.8**	**24.0**	0.0	0.0	0.0	0.0	0.0	0.0
**1461**	1461	allo-Aromadendrene	0.0	0.0	0.0	0.0	0.0	**0.2**	0.0	0.0	0.0	0.0
**1480**	1480	Germacrene D	0.0	**0.9**	0.0	**1.6**	0.0	0.0	0.0	0.0	0.0	0.0
**1493**	1494	Viridiflorene	0.0	0.0	0.0	0.0	0.0	**0.6**	0.0	0.0	0.0	0.0
**1494**	1495	Bicyclogermacrene	0.0	0.0	0.0	**1.2**	0.0	0.0	0.0	0.0	0.0	0.0
**1508**	1511	*trans*-α-Farnesene	0.0	0.0	0.0	**1.0**	0.0	0.0	0.0	0.0	0.0	0.0
**1524**	1524	δ-Cadinene	0.0	0.0	0.0	0.0	0.0	0.0	**0.5**	0.0	0.0	0.0

RTI Tab	RTI Exp	Compound	*Laurus nobilis*	*Lavandula angustifolia × L. latifolia*	*Lavandula angustifolia*	*Matricaria chamomilla*	*Melaleuca alternifolia*	*Melaleuca viridiflora*	*Melissa officinalis*	*Mentha × piperita (Leaf)*	*Mentha × piperita (Leaf/Twig)*	*Mentha arvensis*
**1564**	1568	trans-Nerolidol	0.0	0.0	0.0	0.0	0.0	**0.2**	0.0	0.0	0.0	0.0
**1576**	1577	Germacrene D-4-ol	0.0	0.0	0.0	**0.6**	0.0	0.0	0.0	0.0	0.0	0.0
**1581**	1578	Spathulenol	0.0	0.0	0.0	0.0	0.0	0.0	**0.1**	0.0	0.0	0.0
**1590**	1584	Caryophyllene oxide	0.0	0.0	0.0	0.0	0.0	**2.4**	0.0	0.0	0.0	0.0
**1645**	1642	α-Muurolol	0.0	0.0	0.0	**0.7**	0.0	0.0	0.0	0.0	0.0	0.0
**1655**	1656	α-Bisabolol oxide B	0.0	0.0	0.0	**6.4**	0.0	0.0	0.0	0.0	0.0	0.0
**1725**	1728	Chamazulene	0.0	0.0	0.0	**2.6**	0.0	0.0	0.0	0.0	0.0	0.0
**1744**	1749	α-Bisabolol oxide A	0.0	0.0	0.0	**47.0**	0.0	0.0	0.0	0.0	0.0	0.0

Table 5

**RTI Tab**	**RTI Exp**	**Compound**	***Myristica fragrans***	***Myrtus communis***	***Ocimum basilicum***	***Origanum majorana***	***Origanum vulgare***	***Pelargonium graveolens***	***Pimpinella anisum***	***Pinus mugo***	***Pinus sylvestris***	***Piper nigrum***
**931**	930	α-Thujene	0.0	0.0	0.0	**0.5**	**0.1**	0.0	**0.4**	0.0	0.0	**0.7**
**939**	936	α-Pinene	**23.0**	**15.1**	**0.3**	**0.7**	**1.2**	**0.6**	0.0	**20.7**	**39.3**	**5.5**
**953**	951	Camphene	**0.2**	0.0	0.0	0.0	**0.1**	0.0	0.0	**0.8**	**1.0**	**0.2**
**976**	976	Sabinene	**27.0**	0.0	**0.1**	**5.8**	0.0	0.0	0.0	0.0	0.0	**6.8**
**980**	978	β-Pinene	**13.0**	0.0	**0.4**	**0.4**	**0.3**	**0.4**	0.0	**11.8**	**21.0**	**6.1**
**978**	983	1-Octen-3-ol	0.0	0.0	0.0	0.0	**0.1**	0.0	0.0	0.0	0.0	0.0
**985**	989	6-Methyl-5-hepten-2-one	0.0	0.0	0.0	0.0	0.0	**0.3**	0.0	0.0	0.0	0.0
**991**	992	β-Myrcene	**1.1**	**0.3**	**0.2**	**1.1**	**2.2**	**0.3**	0.0	**11.4**	**1.6**	**1.1**
**1005**	1004	α-Phellandrene	**1.4**	0.0	0.0	**0.3**	**0.2**	0.0	**0.3**	**0.8**	**0.2**	**0.9**
**1011**	1011	Δ-3-Carene	**0.4**	0.0	0.0	0.0	0.0	0.0	**0.3**	**16.5**	**14.1**	**5.9**
**1018**	1018	α-Terpinene	**0.9**	0.0	0.0	**7.0**	**1.0**	0.0	**0.1**	**0.5**	**0.2**	0.0
**1026**	1028	*p*-Cymene	**0.7**	**2.4**	0.0	**4.9**	**12.2**	**0.1**	**0.0**	**2.6**	**0.5**	0.0
**1031**	1031	Limonene	**10.0**	**28.9**	**0.2**	**3.0**	**1.4**	**0.3**	**2.1**	**11.6**	**10.0**	**12.5**
**1031**	1033	β-Phellandrene	0.0	0.0	0.0	0.0	0.0	0.0	0.0	**11.2**	**1.6**	0.0
**1033**	1033	1,8-Cineole	**1.8**	0.0	**3.4**	**0.7**	**0.4**	0.0	0.0	0.0	0.0	0.0
**1050**	1051	*trans*-β-Ocimene	**0.1**	0.0	**1.0**	0.0	0.0	0.0	0.0	0.0	0.0	0.0
**1062**	1061	γ-Terpinene	**4.8**	0.0	0.0	**10.8**	**5.1**	0.0	**0.1**	0.0	**0.1**	**0.2**
**1088**	1089	α-Terpinolene	**0.6**	**0.7**	**0.1**	**2.4**	0.0	0.0	**0.1**	**1.2**	**2.8**	**0.3**
**1098**	1100	Linalool	**0.2**	**13.5**	**0.6**	**34.0**	**1.9**	**6.7**	**0.4**	0.0	0.0	**0.3**
**1111**	1113	*cis*-Rose oxide	0.0	0.0	0.0	0.0	0.0	**0.3**	0.0	0.0	0.0	0.0
**1112**	1114	Endo-fenchol	0.0	0.0	**0.1**	0.0	0.0	0.0	0.0	0.0	0.0	0.0
**1110**	1115	Phenethyl alcohol	0.0	0.0	0.0	0.0	0.0	**0.7**	0.0	0.0	0.0	0.0
**1127**	1130	*trans*-Rose oxide	0.0	0.0	0.0	0.0	0.0	**0.4**	0.0	0.0	0.0	0.0
**1143**	1147	Camphor	0.0	0.0	**0.4**	0.0	0.0	0.0	0.0	0.0	0.0	0.0
**1154**	1157	Menthone	0.0	0.0	0.0	0.0	0.0	**1.1**	0.0	0.0	0.0	0.0
**1165**	1165	Borneol	0.0	0.0	0.0	0.0	**0.3**	0.0	0.0	0.0	0.0	0.0
**1164**	1167	Isomenthone	0.0	0.0	0.0	0.0	0.0	**5.1**	0.0	0.0	0.0	0.0
**1177**	1178	4-Terpineol	**6.7**	0.0	**0.1**	**17.7**	**0.3**	0.0	**0.1**	0.0	0.0	**0.4**
**1189**	1191	α-Terpineol	**0.2**	**2.1**	**0.2**	**3.5**	0.0	**0.6**	0.0	0.0	**0.2**	0.0
**1195**	1199	Estragole	0.0	0.0	**88.4**	0.0	0.0	0.0	**1.7**	0.0	0.0	0.0
**1228**	1232	Citronellol	0.0	0.0	0.0	0.0	0.0	**34.6**	0.0	0.0	0.0	0.0
**1235**	1237	Thymol methyl ether	0.0	0.0	0.0	0.0	0.0	0.0	0.0	**0.6**	0.0	0.0

RTI Tab	RTI Exp	Compound	*Myristica fragrans*	*Myrtus communis*	*Ocimum basilicum*	*Origanum majorana*	*Origanum vulgare*	*Pelargonium graveolens*	*Pimpinella anisum*	*Pinus mugo*	*Pinus sylvestris*	*Piper nigrum*
**1240**	1244	Neral	0.0	0.0	0.0	0.0	0.0	**0.5**	0.0	0.0	0.0	0.0
**1252**	1244	*p*-Anisaldehyde	0.0	0.0	0.0	0.0	0.0	0.0	**0.2**	0.0	0.0	0.0
**1251**	1255	*cis*-Anethol	0.0	0.0	0.0	0.0	0.0	0.0	**0.1**	0.0	0.0	0.0
**1255**	1258	Geraniol	0.0	0.0	0.0	0.0	0.0	**18.5**	0.0	0.0	0.0	0.0
**1257**	1264	Linalyl acetate	0.0	**5.0**	0.0	0.0	0.0	0.0	0.0	0.0	0.0	0.0
**1270**	1273	Geranial	0.0	0.0	0.0	0.0	0.0	**0.7**	0.0	0.0	0.0	0.0
**1275**	1275	Citronellyl formate	0.0	0.0	0.0	0.0	0.0	**9.5**	0.0	0.0	0.0	0.0
**1285**	1287	Bornyl acetate	0.0	0.0	**0.1**	0.0	0.0	0.0	0.0	**3.7**	**3.0**	0.0
**1285**	1288	Safrole	**0.7**	0.0	0.0	0.0	0.0	0.0	0.0	0.0	0.0	0.0
**1290**	1292	Thymol	0.0	0.0	0.0	0.0	**0.2**	0.0	0.0	0.0	0.0	0.0
**1298**	1299	Carvacrol	0.0	0.0	0.0	0.0	**67.4**	0.0	0.0	0.0	0.0	0.0
**1283**	1301	*trans*-Anethol	0.0	0.0	0.0	0.0	0.0	0.0	**92.2**	0.0	0.0	0.0
**1300**	1305	Geranyl formate	0.0	0.0	0.0	0.0	0.0	**4.9**	0.0	0.0	0.0	0.0
**1322**	1325	Mirtenyl acetate	0.0	**13.6**	0.0	0.0	0.0	0.0	0.0	0.0	0.0	0.0
**1339**	1339	δ-Elemene	0.0	0.0	0.0	0.0	0.0	0.0	0.0	0.0	0.0	**1.9**
**1350**	1343	α-Terpinyl acetate	0.0	**1.2**	0.0	0.0	0.0	0.0	0.0	0.0	0.0	0.0
**1351**	1351	α-Cubebene	0.0	0.0	0.0	0.0	0.0	0.0	0.0	0.0	0.0	**0.4**
**1356**	1360	Eugenol	**0.5**	0.0	0.0	0.0	0.0	0.0	0.0	0.0	0.0	0.0
**1372**	1377	α-Copaene	0.0	0.0	0.0	0.0	0.0	**0.4**	0.0	0.0	0.0	**6.4**
**1384**	1385	α-Bourbonene	0.0	0.0	0.0	0.0	0.0	**0.7**	0.0	0.0	0.0	0.0
**1383**	1386	Geranyl acetate	0.0	**3.7**	0.0	0.0	0.0	0.0	0.0	0.0	0.0	0.0
**1390**	1390	β-Cubebene	0.0	0.0	0.0	0.0	0.0	0.0	0.0	0.0	0.0	**0.5**
**1391**	1393	β-Elemene	0.0	0.0	0.0	0.0	0.0	0.0	0.0	0.0	0.0	**0.9**
**1401**	1407	Methyl eugenol	0.0	0.0	**0.3**	0.0	0.0	0.0	0.0	0.0	0.0	0.0
**1418**	1419	*trans*-β-Caryophyllene	0.0	**1.2**	**0.1**	**1.7**	**4.7**	**0.9**	**0.2**	**5.5**	**3.6**	**34.1**
**1436**	1437	α-*trans*-Bergamotene	0.0	0.0	**2.3**	0.0	0.0	0.0	0.0	0.0	0.0	0.0
**1454**	1455	α-Humulene	0.0	**1.4**	0.0	0.0	0.0	0.0	0.0	**0.4**	**0.2**	**2.0**
**1480**	1480	Germacrene D	0.0	0.0	0.0	0.0	0.0	**0.8**	0.0	0.0	0.0	0.0
**1494**	1495	Bicyclogermacrene	0.0	0.0	0.0	**0.7**	0.0	0.0	0.0	0.0	0.0	0.0
**1509**	1510	β-Bisabolene	0.0	0.0	0.0	0.0	0.0	0.0	**0.1**	0.0	0.0	**2.7**
**1513**	1514	*trans*-γ-Cadinene	0.0	0.0	**0.3**	0.0	0.0	0.0	0.0	**0.6**	0.0	0.0
**1524**	1524	δ-Cadinene	0.0	0.0	0.0	0.0	0.0	**0.4**	0.0	0.0	0.0	**2.0**
**1520**	1526	Myristicin	**4.3**	0.0	0.0	0.0	0.0	0.0	0.0	0.0	0.0	0.0

RTI Tab	RTI Exp	Compound	*Myristica fragrans*	*Myrtus communis*	*Ocimum basilicum*	*Origanum majorana*	*Origanum vulgare*	*Pelargonium graveolens*	*Pimpinella anisum*	*Pinus mugo*	*Pinus sylvestris*	*Piper nigrum*
**1529**	1532	Citronellyl-N-butyrate	0.0	0.0	0.0	0.0	0.0	**0.4**	0.0	0.0	0.0	0.0
**1562**	1565	Geranyl-N-butyrate	0.0	0.0	0.0	0.0	0.0	**2.1**	0.0	0.0	0.0	0.0
**1581**	1578	Spathulenol	0.0	0.0	0.0	0.0	0.0	0.0	0.0	0.0	0.0	**2.2**
**1619**	1619	10-epi-γ-Eudesmol	0.0	0.0	0.0	0.0	0.0	**3.4**	0.0	0.0	0.0	0.0
**1677**	1681	Foeniculin	0.0	0.0	0.0	0.0	0.0	0.0	**0.7**	0.0	0.0	0.0
**1701**	1704	Geranyl tiglate	0.0	0.0	0.0	0.0	0.0	**1.0**	0.0	0.0	0.0	0.0

Table 6

**RTI Tab**	**RTI Exp**	**Compound**	***Pogostemon cablin***	***Rosa × damascena***	***Salvia officinalis***	***Salvia sclarea***	***Santalum album***	***Syzygium aromaticum***	***Thuja occidentalis***	***Thymus vulgaris***	***Verbena officinalis***	***Zingiber officinale***
**931**	930	α-Thujene	0.0	0.0	**0.6**	0.0	0.0	0.0	0.0	0.0	0.0	0.0
**939**	936	α-Pinene	0.0	**0.1**	**4.6**	0.0	**0.6**	0.0	**1.5**	**1.0**	**3.1**	**2.3**
**953**	951	Camphene	0.0	0.0	**4.8**	0.0	0.0	0.0	**3.0**	**0.6**	**0.2**	**5.1**
**976**	976	Sabinene	0.0	0.0	**0.2**	0.0	**0.6**	0.0	**2.0**	0.0	**0.6**	0.0
**980**	978	β-Pinene	**0.1**	0.0	**2.3**	0.0	**0.4**	0.0	**0.3**	0.0	**3.4**	**0.3**
**985**	989	6-Methyl-5-hepten-2-one	0.0	0.0	0.0	0.0	0.0	0.0	0.0	0.0	**0.9**	**0.6**
**991**	992	β-Myrcene	0.0	**0.1**	**0.9**	**0.7**	0.0	0.0	**2.0**	**1.3**	**0.6**	**0.8**
**1005**	1004	α-Phellandrene	0.0	**0.1**	0.0	0.0	0.0	0.0	0.0	0.0	0.0	**0.2**
**1018**	1018	α-Terpinene	0.0	0.0	0.0	0.0	0.0	0.0	0.0	**1.1**	0.0	0.0
**1026**	1028	*p*-Cymene	0.0	0.0	**0.7**	0.0	0.0	0.0	**0.8**	**21.9**	0.0	0.0
**1031**	1031	Limonene	0.0	0.0	**1.5**	**0.2**	**0.1**	0.0	0.0	**0.7**	**12.7**	0.0
**1031**	1033	β-Phellandrene	0.0	0.0	0.0	0.0	0.0	0.0	0.0	0.0	0.0	**6.9**
**1033**	1033	1,8-Cineole	0.0	0.0	**11.4**	0.0	0.0	0.0	**1.8**	**0.2**	**0.5**	0.0
**-**	1038	Benzyl alcohol	0.0	**0.7**	0.0	0.0	0.0	0.0	0.0	0.0	0.0	0.0
**1040**	1041	*cis*-β-Ocimene	0.0	0.0	0.0	**0.3**	0.0	0.0	0.0	0.0	0.0	0.0
**1041**	1045	Salicylaldehyde	0.0	0.0	0.0	0.0	0.0	0.0	0.0	0.0	0.0	0.0
**1050**	1051	*trans*-β-Ocimene	0.0	0.0	0.0	**0.6**	0.0	0.0	0.0	0.0	0.0	0.0
**1062**	1061	γ-Terpinene	0.0	0.0	**1.9**	0.0	0.0	0.0	**14.0**	**9.4**	0.0	0.0
**1087**	1090	Fenchone	0.0	0.0	0.0	0.0	0.0	0.0	**13.5**	0.0	0.0	0.0
**1088**	1089	α-Terpinolene	0.0	0.0	**0.7**	**0.2**	0.0	0.0	0.0	0.0	0.0	**0.2**
**1098**	1100	Linalool	0.0	**0.1**	0.0	**22.0**	0.0	0.0	0.0	**4.0**	**0.9**	**0.3**
**1102**	1107	α-Thujone	0.0	0.0	**22.5**	0.0	0.0	0.0	**39.3**	0.0	0.0	0.0
**1110**	1115	Phenethyl alcohol	0.0	**75.0**	0.0	0.0	0.0	0.0	0.0	0.0	0.0	0.0
**1114**	1118	β-Thujone	0.0	0.0	**6.2**	0.0	0.0	0.0	**6.8**	0.0	0.0	0.0
**1143**	1147	Camphor	0.0	0.0	**18.5**	0.0	0.0	0.0	**19.8**	0.0	0.0	0.0
**1153**	1158	Citronellal	0.0	0.0	0.0	0.0	0.0	0.0	0.0	0.0	**5.9**	0.0
**1165**	1165	Borneol	0.0	0.0	**4.4**	0.0	0.0	0.0	**0.4**	0.0	0.0	**1.3**
**1177**	1178	4-Terpineol	0.0	0.0	**0.7**	0.0	0.0	0.0	**2.3**	**0.9**	0.0	0.0
**1189**	1191	α-Terpineol	0.0	0.0	0.0	**3.0**	0.0	0.0	0.0	0.0	**0.2**	**0.7**
**1204**	1207	*N*-Decanal	0.0	0.0	0.0	0.0	0.0	0.0	0.0	0.0	0.0	**0.3**
**1228**	1232	Citronellol	0.0	**11.0**	0.0	0.0	0.0	0.0	0.0	0.0	**1.4**	0.0

RTI Tab	RTI Exp	Compound	*Pogostemon cablin*	*Rosa × damascena*	*Salvia officinalis*	*Salvia sclarea*	*Santalum album*	*Syzygium aromaticum*	*Thuja occidentalis*	*Thymus vulgaris*	*Verbena officinalis*	*Zingiber officinale*
**1228**	1232	Nerol	0.0	0.0	0.0	**0.5**	0.0	0.0	0.0	0.0	0.0	0.0
**1235**	1237	Thymol methyl ether	0.0	0.0	0.0	0.0	0.0	0.0	0.0	0.0	0.0	0.0
**1240**	1244	Neral	0.0	0.0	0.0	0.0	0.0	0.0	0.0	0.0	**26.4**	0.0
**1244**	1244	Carvacrol methyl ether	0.0	0.0	0.0	0.0	0.0	0.0	0.0	**0.2**	0.0	0.0
**1255**	1258	Geraniol	0.0	**6.0**	0.0	0.0	0.0	0.0	0.0	0.0	**2.8**	0.0
**1257**	1264	Linalyl acetate	0.0	0.0	0.0	**62.5**	0.0	0.0	0.0	0.0	0.0	0.0
**1270**	1273	Geranial	0.0	**0.1**	0.0	0.0	0.0	0.0	**2.6**	0.0	**34.6**	**0.2**
**1285**	1287	Bornyl acetate	0.0	0.0	**2.3**	0.0	0.0	0.0	0.0	0.0	0.0	0.0
**1290**	1292	Thymol	0.0	0.0	0.0	0.0	0.0	0.0	0.0	**50.1**	0.0	0.0
**1298**	1299	Carvacrol	0.0	0.0	0.0	0.0	0.0	0.0	0.0	**3.2**	0.0	0.0
**1354**	1356	Citronellyl acetate	0.0	0.0	0.0	0.0	0.0	0.0	0.0	0.0	**0.4**	0.0
**1356**	1360	Eugenol	0.0	**1.4**	0.0	0.0	0.0	**82.0**	0.0	0.0	0.0	0.0
**1365**	1369	Neryl acetate	0.0	0.0	0.0	**1.6**	0.0	0.0	0.0	0.0	0.0	**0.6**
**1372**	1377	α-Copaene	0.0	0.0	0.0	**0.4**	**0.2**	0.0	0.0	0.0	0.0	**0.6**
**1380**	1381	β-Patchoulene	**2.5**	0.0	0.0	0.0	0.0	0.0	0.0	0.0	0.0	0.0
**1383**	1386	Geranyl acetate	0.0	0.0	0.0	**2.1**	0.0	0.0	0.0	0.0	**0.2**	**1.4**
**1391**	1393	β-Elemene	**1.0**	0.0	0.0	0.0	0.0	0.0	0.0	0.0	0.0	**1.1**
**1401**	1407	Methyl eugenol	0.0	**0.7**	0.0	0.0	0.0	**0.0**	0.0	0.0	0.0	0.0
**1409**	1411	α-Cedrene	0.0	0.0	0.0	0.0	**4.7**	0.0	0.0	0.0	0.0	**0.4**
**1418**	1419	*trans*-β-Caryophyllene	**2.9**	**0.2**	**5.9**	**2.3**	**20.3**	**9.1**	**1.6**	**1.4**	**1.2**	0.0
**1429**	1431	*cis*-Tujopsene	0.0	0.0	0.0	0.0	**4.4**	0.0	0.0	0.0	0.0	0.0
**1436**	1437	α-*trans*-Bergamotene	0.0	0.0	0.0	**0.3**	0.0	0.0	0.0	0.0	0.0	0.0
**1439**	1442	α-Guaiene + γ-Patchoulene	**13.2**	0.0	0.0	0.0	0.0	0.0	0.0	0.0	0.0	0.0
**1449**	1449	epi-β-Santalene	0.0	0.0	0.0	0.0	**0.4**	0.0	0.0	0.0	0.0	0.0
**1454**	1455	α-Humulene	0.0	**0.1**	**7.2**	**0.3**	**1.9**	**1.1**	0.0	0.0	0.0	0.0
**1456**	1456	α-Patchoulene	**6.9**	0.0	0.0	0.0	0.0	0.0	0.0	0.0	0.0	0.0
**1462**	1462	β-Santalene	0.0	0.0	0.0	0.0	**0.7**	0.0	0.0	0.0	0.0	0.0
**1480**	1480	Germacrene D	0.0	0.0	0.0	**0.4**	0.0	0.0	0.0	0.0	**0.3**	0.0
**1483**	1484	α-Curcumene	0.0	0.0	0.0	0.0	**0.4**	0.0	0.0	0.0	0.0	**9.4**
**1499**	1498	α-Muurolene	0.0	0.0	0.0	0.0	**0.9**	0.0	0.0	0.0	0.0	0.0
**1495**	1499	α-Zingiberene	0.0	0.0	0.0	0.0	0.0	0.0	0.0	0.0	0.0	**30.4**

RTI Tab	RTI Exp	Compound	*Pogostemon cablin*	*Rosa × damascena*	*Salvia officinalis*	*Salvia sclarea*	*Santalum album*	*Syzygium aromaticum*	*Thuja occidentalis*	*Thymus vulgaris*	*Verbena officinalis*	*Zingiber officinale*
**1502**	1505	Cuparene	0.0	0.0	0.0	0.0	**0.9**	0.0	0.0	0.0	0.0	0.0
**1505**	1508	β-Bulnesene	**19.7**	0.0	0.0	0.0	0.0	0.0	0.0	0.0	0.0	0.0
**1509**	1510	β-Bisabolene	0.0	0.0	0.0	**0.2**	0.0	0.0	0.0	0.0	0.0	**8.0**
**1513**	1514	*trans*-γ-Cadinene	0.0	0.0	0.0	0.0	0.0	0.0	0.0	0.0	0.0	**1.0**
**1524**	1524	δ-Cadinene	0.0	0.0	0.0	**0.2**	**1.4**	0.0	0.0	0.0	**0.3**	0.0
**1524**	1525	β-Sesquifellandrene	0.0	0.0	0.0	0.0	0.0	0.0	0.0	0.0	0.0	**12.6**
**1524**	1533	Eugenyl acetate	0.0	0.0	0.0	0.0	0.0	**7.1**	0.0	0.0	0.0	0.0
**1533**	1534	*trans*-γ-Bisabolene	0.0	0.0	0.0	0.0	0.0	0.0	0.0	0.0	0.0	**0.3**
**1542**	1542	Selina-3,7(11)-diene	0.0	0.0	0.0	0.0	**0.4**	0.0	0.0	0.0	0.0	0.0
**1549**	1551	Elemol	0.0	0.0	0.0	0.0	**1.9**	0.0	0.0	0.0	0.0	0.0
**1556**	1557	Germacrene B	0.0	0.0	0.0	0.0	0.0	0.0	0.0	0.0	0.0	**0.4**
**1564**	1568	trans-Nerolidol	0.0	0.0	0.0	0.0	0.0	0.0	0.0	0.0	0.0	**0.6**
**1581**	1578	Spathulenol	0.0	0.0	0.0	**0.2**	0.0	**0.3**	0.0	0.0	0.0	0.0
**1596**	1599	α-Cedrol	0.0	0.0	0.0	0.0	**3.2**	0.0	0.0	0.0	0.0	0.0
**1619**	1619	10-epi-γ-Eudesmol	0.0	0.0	0.0	0.0	**1.2**	0.0	0.0	0.0	0.0	0.0
**1630**	1633	γ-Eudesmol	0.0	0.0	0.0	0.0	**1.6**	0.0	0.0	0.0	0.0	0.0
**1655**	1654	Valerianol	0.0	0.0	0.0	0.0	**2.1**	0.0	0.0	0.0	0.0	0.0
**1658**	1659	7-epi-α-Eudesmol	0.0	0.0	0.0	0.0	**1.8**	0.0	0.0	0.0	0.0	0.0
**1659**	1661	Patchouli alcohol	**33.4**	0.0	0.0	0.0	0.0	0.0	0.0	0.0	0.0	0.0
**1678**	1679	*cis*-α-Santalol	0.0	0.0	0.0	0.0	**18.8**	0.0	0.0	0.0	0.0	0.0
**1693**	1693	*trans*-α-Bergamotol	0.0	0.0	0.0	0.0	**2.7**	0.0	0.0	0.0	0.0	0.0
**1704**	1705	epi-β-Santalolo	0.0	0.0	0.0	0.0	**1.8**	0.0	0.0	0.0	0.0	0.0
**1720**	1720	*cis*-β-Santalolo	0.0	0.0	0.0	0.0	**7.8**	0.0	0.0	0.0	0.0	0.0
**1727**	1725	*cis*-nuciferol	0.0	0.0	0.0	0.0	**1.4**	0.0	0.0	0.0	0.0	0.0
**2220**	2216	Sclareol	0.0	0.0	0.0	**0.9**	0.0	0.0	0.0	0.0	0.0	0.0
